# Supplementary material for: A Multimodal Intervention for Prevention of Overweight and Obesity in Schoolchildren. A Protocol Study “PREVIENE-CÁDIZ”
Source: Int J Environ Res Public Health. 2021 Feb 8;18(4):1622. doi: 10.3390/ijerph18041622 (PMC7914756; doi:10.3390/ijerph18041622)
Supplement: Supplementary file 1 [file ijerph-18-01622-s001.zip › Document S10.pdf]

Cuestionario - Programa *PREVIENE CÁDIZ*.

Directores/as – Equipos directivos - Docentes

Gracias por participar en este proyecto. Su opinión es muy importante.

Hay 6 preguntas en la encuesta.

Nombre del centro

Localidad

Persona que responde la encuesta:

Director/a del centro						
Profesor/a de la línea		Indicar la línea	1	2	3	4
Profesor/a de Educación Física						
Otra persona		Indicar quien:				

1. ¿Se ha desarrollado en su centro este año el programa "Creciendo en salud"? Sí ☐ No ☐ No lo sé ☐

2. En caso negativo ¿nos podría indicar cuales son las causas de no haberlo desarrollado?

3. En caso afirmativo, indicar qué líneas del mismo se han desarrollado: (en 3er curso)

Educación emocional	
Estilos de vida Saludable	
Autocuidados y Accidentalidad	
Uso Positivo de las Tecnologías de la Información y la Comunicación	
Prevención del Consumo de Sustancias Adictivas	

4. En el caso de que se haya desarrollado alguna/s de las líneas anteriores, indicar qué bloques temáticos se han trabajado: (en 3er curso)

Educación emocional.	
- Conciencia Emocional.	
- Regulación Emocional.	
- Autonomía Emocional.	
- Competencia Social.	
- Competencias para la Vida y el Bienestar.	
Estilos de vida Saludable.	
- Actividad Física.	
- Alimentación Equilibrada.	
Autocuidados y Accidentalidad.	
- Autocuidados	
- Accidentalidad:	
○ Educación Vial	
○ Seguridad en el Hogar	

Uso Positivo de las Tecnologías de la Información y la Comunicación	
- Estilos de Vida Saludable en una Sociedad digital.	
- Adicciones a las TIC.	
- Buenas Prácticas y Recomendaciones.	
Prevención del Consumo de Sustancias Adictivas.	
- Hábitos que contribuyen a una vida sana.	
- Consumo de Alcohol y Tabaco.	
- Publicidad y Drogas: mitos y creencias.	

5. ¿Disponéis en el Centro de un equipo de Promoción y Educación para la Salud? (Consejo de Salud Escolar o similar, grupo de personas encargados de estas actividades) Si ☐ No ☐

En caso afirmativo,

¿Quién lo ha coordinado?____

¿Quién lo compone? (indicar las personas que lo componen, sus cargos y/o colectivos a los que representan, no es necesario indicar nombres y apellidos)

6. Actividades realizadas este curso académico en el centro educativo con el alumnado de 3º curso, relacionadas con la alimentación y/o el ejercicio físico saludables.

Plan Escolar de Consumo de Frutas y Hortalizas (reparto de frutas / hortalizas)	
Desayuno saludable.	
Concurso de recetario infantil.	
Huertos escolares.	
Frutibus	
Otras:	

Indicar qué otras actividades se han realizado:

Muchas gracias por completar el cuestionario.
