# Supplementary material for: A Multimodal Intervention for Prevention of Overweight and Obesity in Schoolchildren. A Protocol Study “PREVIENE-CÁDIZ”
Source: Int J Environ Res Public Health. 2021 Feb 8;18(4):1622. doi: 10.3390/ijerph18041622 (PMC7914756; doi:10.3390/ijerph18041622)

Document S1

1. Summary of the didactic guide applied in the intervention of the study

PREVIENE

Programa de "Alimentación y Actividad Física Saludables"

JUNTA DE ANDALUCIA

CRÉDITOS

Material elaborado por los equipos del proyecto de investigación:
"Prevención de la obesidad infantil en escolares. Efectos de una intervención multicomponente" de la Escuela Andaluza de Salud Pública y la Agencia de Salud Pública de Bracelona, con la colaboración de la Facultad del Deporte y el Centro del Profesorado de Granada.

Coordinación • Carmen Lineros González

Autoría
- Carmen Lineros González
- Raquel Carrasco Campos
- Olga Leralta Piñán
- Carlos Belda Grindley
- Ezequiel García Navarro
- Pablo Tercedor Sánchez
- Isaac Pérez López
- Manuel Delgado Fernández
- Mariano Hernán García

El material ha sido pilotado y validado por profesorado de centros educativos de Granada:
- Lorena Delgado Ocón, C.E.I.P. Federico García Lorca, Fuente Vaqueros
- Lisardo Eugenio Domingo Blanco, C.E.I.P. Sagrado Corazón de Jesús, Alhendín
- Manuel Jiménez López, C.E.I.P. Virgen de la Paz, Otura
- José Eduardo Linares Cuesta, C.E.I.P. Ntra. Sra. de la Salud, Itrabo
- Manuel José Martínez Rivas, C.E.I.P. Ruiz del Peral, Guadix
- Ámparo Morales Molina, C.E.I.P. Profesor Tierno Galván, Pulianas
- Santiago Moreno Muñoz, C.E.I.P. San Andrés, Montejícar
- Dominica Mudarra Mudarra, C.E.I.P. Ntra. Sra. de las Angustias, Huétor-Vega
- Ana Pastora Ortiz Ruiz, C.E.I.P. Manuel de Falla, Peligros
- Tomás Duarte Periago, C.D.P. San Juan de Ávila, La Caleta-Guardia

Con la colaboración del alumnado de la Facultad del Deporte de Granada:
Clara Fernández Martín, Dolores Torres Padua y María Victoria Cáceres Quiñonero.

Granada, 2016

Ediciones 2015 / 2016 • Escuela Andaluza de Salud Pública.
Proyecto de Investigación PI-0652/2012
Consejería de Igualdad, Salud y Políticas Sociales

Edición 2018 • "Prevención del sobrepeso y la obesidad infantil en escolares de la provincia de Cádiz. Estudio cuasiexperimental de la efectividad de una intervención multicomponente".
Número de expediente: PI-0007-2017. IP: Amelia Rodríguez Martín
Centro de Investigación: Universidad de Cádiz.

Ilustraciones • Rubén Garrido

Diseño y maquetación • Catálogo S.L.

Material adaptado del Proyecto de Prevención de la Obesidad Infantil de la Agencia de Salud Pública de Barcelona. Nuestro agradecimiento a Carles Ariza Cardenal y su equipo.

ÍNDICE

1 EL PROGRAMA

Introducción 4
1 • Destinatarios 8
2 • Objetivos 8
3 • Desarrollo de la intervención 8
3.1 • Funciones del profesorado 9
3.2 • Papel del centro educativo 10
3.3 • Papel de las familias 11
3.4 • Actividades con el alumnado 11
4 • Resumen de la intervención 13
5 • Seguimiento y evaluación 14
6 • Cómo contribuye el programa al desarrollo de las competencias 16
7 • Bibliografía 18

2 SESIONES DIDÁCTICAS 21

Sesión 1 • Nuestro crecimiento 22
Sesión 2 • Valorémonos 26
Sesión 3 • Nuestra digestión 29
Sesión 4 • Los grupos de alimentos 32
Sesión 5 • Los nutrientes y sus funciones 37
Sesión 6 • La pirámide de la alimentación y el ejercicio 41
Sesión 7 • El mejor desayuno 45
Sesión 8 • Actividad física y descanso 48
Sesión 9 • Recapitulación y síntesis 51
Recreos Activos 55
Anexo: Registro de actividad de los Recreos Activos 57

3 PREVIENE. ACTIVIDADES PARA EL ALUMNADO 59

Sesión 1 • Nuestro crecimiento 60
Sesión 2 • Valorémonos 64
Sesión 3 • Nuestra digestión 66
Sesión 4 • Los grupos de alimentos 68
Sesión 5 • Los nutrientes y sus funciones 72
Sesión 6 • La pirámide de la alimentación y el ejercicio 77
Sesión 7 • El mejor desayuno 78
Sesión 8 • Actividad física y descanso 84

4 ALIMENTACIÓN Y ACTIVIDAD FÍSICA SALUDABLES EN FAMILIA 89

Introducción 90
• Nuestro crecimiento 92
• Nuestra digestión 94
• La pirámide de la alimentación y el ejercicio 95
• El mejor desayuno 96
• Actividad física y descanso 98

SESIONES DIDÁCTICAS

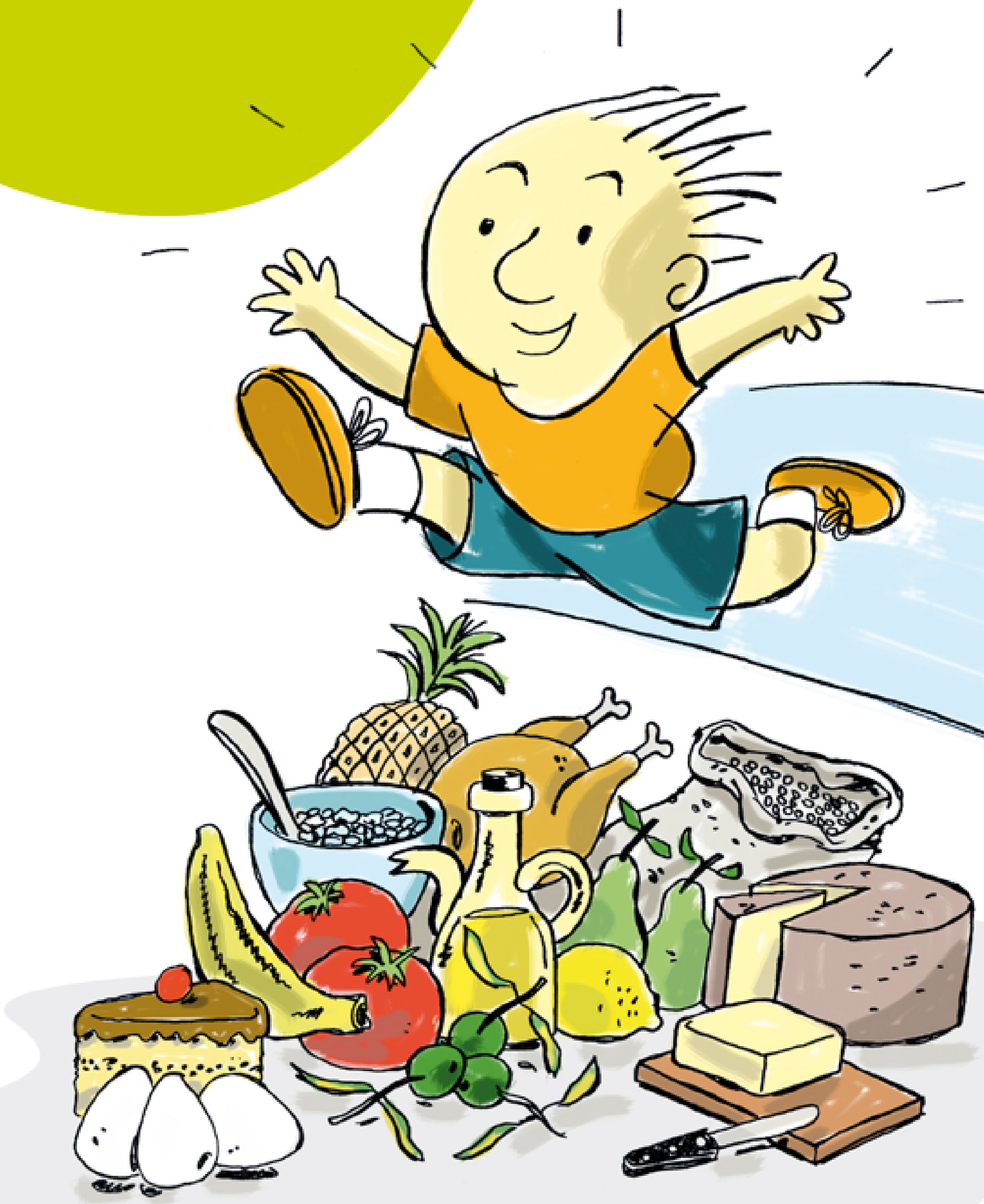

Sesión 1 Nuestro crecimiento

1 Resumen de la sesión

Comparar fotos personales de diferentes edades fijándose que el peso y la altura han ido cambiando a lo largo del tiempo. Incorporar actividades físicas que favorezcan el reconocimiento y aceptación del propio cuerpo y el de los demás.

2 Objetivo

Reconocer el peso y la altura como expresión del crecimiento.

3 Ideas clave

- El peso y la altura van variando con el crecimiento.
- Una alimentación saludable influye favorablemente en el peso y la altura.
- El ejercicio físico saludable influye favorablemente en el crecimiento.
- Otros aspectos (genéticos, ambientales, económicos) pueden influir en el crecimiento de cada persona.

4 Actividades de aprendizaje

4.1. EN EL AULA

Unos días antes de realizar la actividad en el aula, se entrega la ficha para la familia y se informa sobre los materiales que deben llevar a clase: fotos de diferentes momentos de su crecimiento (p.ej. a los pocos días de nacer, a los dos o tres años y una actual). El alumnado pedirá a su familia las fotos y también información sobre el peso y la altura aproximados de cada uno de los momentos fotografiados (véase la ficha para la familia). A medida en que el alumnado va trayendo las fotos al aula, se van colocando en un mural en el aula. Se recomienda que debajo de cada foto figuren su nombre y la edad, y el peso y altura correspondiente a cada foto. Tener en cuenta que puede haber alumnado que no aporte fotos.

Sesión 2

Valorémonos

1 Resumen de la sesión

Se realizan actividades físicas destinadas a valorar rasgos positivos del aspecto físico de cada compañero y compañera de clase.

2 Objetivo

Reconocer y aceptar el aspecto físico propio y el de los demás.

3 Ideas clave

- La valoración del aspecto físico de cada persona depende sobre todo de la valoración de uno mismo y de la que tienen los demás.
- Todos y todas tenemos aspectos físicos positivos y otros mejorables.
- Todos y todas somos diferentes y podemos disfrutar de las diferencias.
- Hay que respetar el aspecto físico propio y el de los demás, tal como queremos que respeten el nuestro.

4 Actividades de aprendizaje

4.1. EN EL AULA

Se organiza la sesión con grupos homogéneos en base a perímetros, peso y altura. Se realizan actividades dirigidas a valorar mediante la actividad física las diferencias individuales mediante diferentes formas de desplazamiento y transporte. Tras la vivencia de la actividad el alumnado anota rasgos positivos de los demás en una ficha (véase la ficha para el alumnado).

Con el nombre en la ficha, el alumnado sabrá sobre quién ha de escribir el rasgo positivo del aspecto físico. Todas las hojas deben ir pasando por el alumnado para conseguir

Sesión 3

Nuestra digestión

1 Resumen sesión

Completar el aparato digestivo con los nombres de las diferentes partes indicando la función de cada una.

2 Objetivo

- Conocer el aparato digestivo y sus funciones.
- Practicar hábitos de higiene relacionados con la alimentación.

3 Ideas clave

1. La digestión comienza en la boca. Es importante masticar bien los alimentos para que se mezclen con la saliva y puedan empezar a ser digeridos.
2. La higiene bucal es muy importante. Hay que cepillarse los dientes después de cada comida.
3. Es aconsejable comer con tranquilidad.
4. Es recomendable reposar al menos unos 20 minutos después de comer para no interrumpir el proceso digestivo.
5. Comer alimentos como frutas, verduras y alimentos integrales ayuda a los intestinos a funcionar mejor.
6. Importancia de lavarse las manos antes y después de comer.

4 Actividades de aprendizaje

4.1. EN EL AULA

Como previo a la actividad en el aula el alumnado ha elaborado carteles con las partes del aparato digestivo (se sugiere aprovechar la clase de plástica). Con la base fundamental de practicar actividad física, el alumnado se dirige alternativamente a los pósters pegados a la pared, previamente elaborados, con dibujos o nombres de las once partes

Sesión 4

Los grupos de alimentos

1 Resumen de la sesión

La sesión propuesta tiene dos partes: actividad en la que se combina agrupaciones y dispersiones en base al trabajo con grupos de alimentos, dieta completa, completando alimentos, caloría vacía, hidratando el cuerpo. Y, para terminar, clasificar los envases que hayan llevado según el grupo de alimentos al que pertenecen.

2 Objetivos

Conocer los diferentes grupos de alimentos y diferenciarlos.

3 Ideas clave

1. Los alimentos se pueden clasificar en los siguientes grupos: lácteos, carne-pescados-huevos, féculas (cereales, patatas, legumbres), frutas-verduras, grasas, azúcares. Incluir el agua como elemento esencial en la alimentación.
2. Los alimentos también se pueden clasificar según su origen: animal, vegetal, mineral.
3. En las tiendas de alimentación podemos encontrar diferentes presentaciones de cada uno de los alimentos: frescos, congelados, cocinados, etc.

4 Actividades de aprendizaje

4.1. EN EL AULA

Unos días antes de esta sesión, el profesorado pide al alumnado que traiga de casa diferentes envases alimentarios (botellas de leche y otras bebidas, envase de yogur, bolsas de productos congelados, etc.), dibujos o fotografías de alimentos y actividades físicas. Este material servirá para construir, en sesiones pósteriores, una pirámide de la alimentación y la actividad física.

Sesión 5

Los nutrientes y sus funciones

1 Resumen de la sesión

La sesión consiste en relacionar los nutrientes y sus funciones mediante actividades físicas en las que impera el juego y la cooperación entre el alumnado.

2 Objetivos

Conocer los nutrientes y sus funciones.

3 Ideas clave

1. Los nutrientes son sustancias indispensables para la vida que el organismo aprovecha de los alimentos.
2. En cada alimento predomina un nutriente u otro, y por eso tiene tanta importancia seguir una alimentación variada y equilibrada.
3. Los nutrientes se pueden clasificar según la función principal que tienen en:
   - **Formadores o plásticos:** construyen los músculos y los huesos y renuevan las partes que crecen constantemente.
   - **Energéticos:** proporcionan la energía necesaria para desarrollar las tareas diarias.
   - **Reguladores:** regulan el funcionamiento del cuerpo.
4. Hay que comer proteínas dos o tres veces al día.
5. Las proteínas pueden ser de origen animal o vegetal.
6. Hay que comer verduras dos o tres veces al día.

4 Actividades de aprendizaje

4.1. EN EL AULA

Utilizando las tarjetas de colores de la sesión 4, se proponen nuevas actividades y tareas:

Sesión 6

La pirámide de la alimentación y el ejercicio

1 Resumen de la sesión

Construir una pirámide de los hábitos saludables (alimentación y ejercicio físico) con los envases, dibujos y fotos de alimentos y actividad física que el alumnado ha aportado desde casa. Diseñar dietas saludables.

2 Objetivos

- Conocer las principales recomendaciones alimentarias y sobre ejercicio físico.
- Mostrar habilidades asociadas a estas recomendaciones.

3 Ideas clave

ALIMENTACIÓN:

- **Dulces, bollería, embutidos, aperitivos (snacks, piscolabis) y carne roja (cerdo, ternera, buey, cordero):** sólo algunas veces al mes.
- **Pescado y marisco:** 3-4 a la semana.
- **Legumbres:** 3-4 a la semana.
- **Huevos:** 3-4 a la semana.
- **Carne blanca (pollo, pavo, conejo):** 3-4 a la semana.
- **Lácteos:** 3 veces al día.
- **Aceite:** 3-5 cucharadas al día.
- **Frutos secos:** 3-7 puñados a la semana.
- **Pan, patatas, arroz, pasta y otros cereales:** 4-6 al día.
- **Verduras, hortalizas y fruta:** 5 al día o más.
- **Agua:** De 4 a 8 vasos al día (1 litro).

Hay diferentes presentaciones de cada alimento y que éste, por tanto, se puede consumir de diferentes maneras. Se sugiere hacer mención a dietas específicas (por ej. comentar si en la familia hay diabéticos, celiácos, etc.) que no incorporan algunos de los alimentos que desde el programa se recomiendan, ya se a por motivos de salud (alergias alimentarias) o por la diversidad cultural y étnica presente en Andalucía.

Incorporar las recomendaciones sobre Actividad física (hacer deporte, actividades dirigidas, caminar, subir escaleras): mínimo, 30 minutos al día.

Sesión 7

El mejor desayuno

1 Resumen actividad

Desarrollo de un taller de desayuno saludable andaluz, donde estén representados los alimentos más importantes para lograr que sea equilibrado, variado, divertido.

2 Objetivos

- Identificar el contenido y la cantidad de un desayuno saludable.
- Experimentar que el desayuno puede ser atractivo y deseable.

3 Ideas clave

1. El desayuno saludable incluye: fruta, lácteos y pan, galletas o cereales.
2. El desayuno se reparte entre lo que comemos en casa por la mañana, y lo que tomamos en la escuela (merienda / tentempié).
3. Es tan importante la clase de alimentos que tomamos para desayunar, como la cantidad de comida.
4. Un buen desayuno tiene como principales ventajas:
    - Recuperar la energía perdida después de haber dormido durante horas sin haber ingerido ningún alimento.
    - Contribuir al equilibrio alimentario y a una asimilación más regular y más eficaz de los nutrientes.
    - Evitar el golpe de cansancio a media mañana.
    - Asegurar una buena forma y mejorar la atención y la eficacia en clase.
    - Controlar el peso, gracias a una alimentación más equilibrada y regular.
    - Favorecer un buen nivel de azúcar en sangre y la mejora del rendimiento.

Sesión 8

Actividad física y descanso

1 Resumen de la sesión

Practicar/simular las distintas recomendaciones de la actividad física, propuestas por la pirámide.

Como contenidos:
Los hábitos de la actividad física y el descanso.
La relación entre actividad física, descanso, alimentación y nutrición.

2 Objetivos

Mejorar los hábitos saludables de actividad y descanso.

3 Ideas clave

1. A los nueve o diez años se debe dormir nueve o diez horas diarias para poder recuperar fuerzas y rendir mejor al día siguiente.
2. El tiempo máximo dedicado a ver la TV, jugar al ordenador o videojuegos y, en general, al uso de pantallas o juegos sedentarios, no debe superar las dos horas diarias. Si un día dedicamos más de dos horas, quiere decir que utilizamos menos tiempo para movernos, y entonces nuestro cuerpo acumula energía que no se quema.
3. Se recomiendan 60 minutos al día de actividad física (juego movido, caminar, bajar y subir escaleras, correr por el patio, parque o jardín, etc.).
4. Para que el ejercicio físico tenga efectos en nuestro cuerpo (refuerce el corazón, fortalezca los músculos y huesos, permita obtener metas comunes en equipo, etc.), es recomendable hacer seis horas a la semana de actividad física (educación física, deporte, juego, actividades de la vida diaria...) (2+2+2).
5. Para rendir de manera equilibrada se necesitan tres elementos:

   a. Actividad física suficiente.
   b. Descanso adecuado.
   c. Una alimentación saludable.

Sesión 9

Recapitulación y síntesis

1 Resumen de la sesión

- Resumen de las ideas clave de las sesiones precedentes.
- Revisión de las propuestas de mejora de hábitos.
- Valoración final y cierre del programa.

2 Objetivos

Revisar las propuestas para integrar la actividad física, el descanso y la alimentación saludables como hábitos.

3 Ideas clave

1. Hay que respetar nuestro propio aspecto físico y también el de los demás, como queremos que hagan con el nuestro.
2. Para rendir de manera equilibrada se necesitan varios elementos:
   - **a.** Actividad física suficiente (30 minutos mínimo al día).
   - **b.** Descanso adecuado (9-10 horas al día de sueño y un máximo de 2 horas al día de actividades con uso de pantallas).
   - **c.** Una alimentación saludable.
   - **d.** Una hidratación saludable basada en agua.
3. Una alimentación saludable debe ser variada (presencia de todos los alimentos) y equilibrada (para obtener la energía adecuada).
4. **RECOMENDACIONES:**
   - **CADA DÍA:** 30 minutos de actividad física, beber un total de 2 litros de agua y comer féculas, cereales, verduras, frutas y lácteos.
   - **ALGUNAS VECES A LA SEMANA:** carnes blancas, huevos, pescado y legumbres.
   - **ALGUNAS VECES AL MES:** carnes rojas, jamón, embutidos y dulces, pastelería y snacks.
5. Un desayuno saludable incluye, entre lo que comemos en casa y lo que tomamos en la escuela: cereales, fruta y lácteos.

2. Cover of the student workbook.

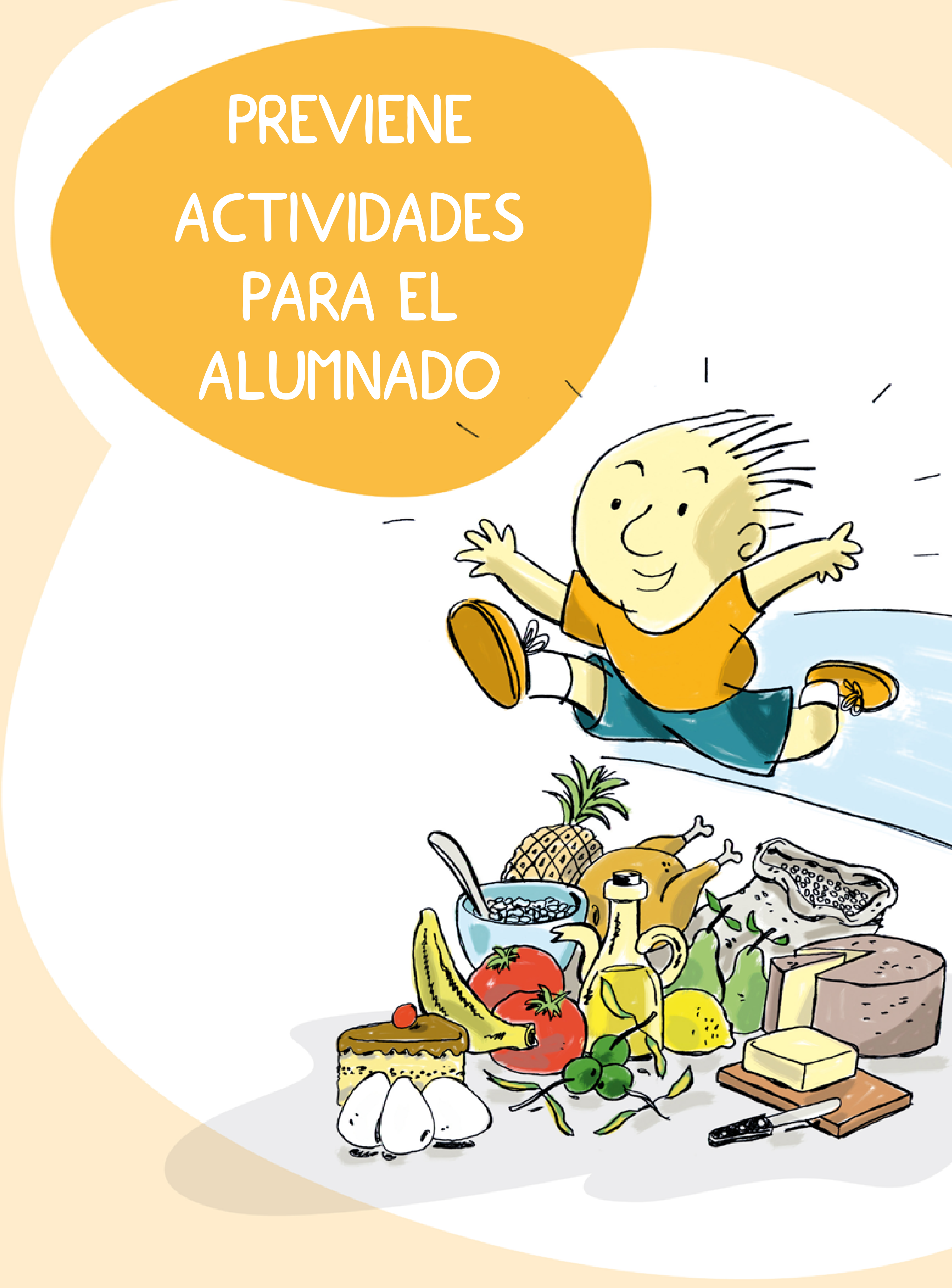

3. Cover of the family workbook.

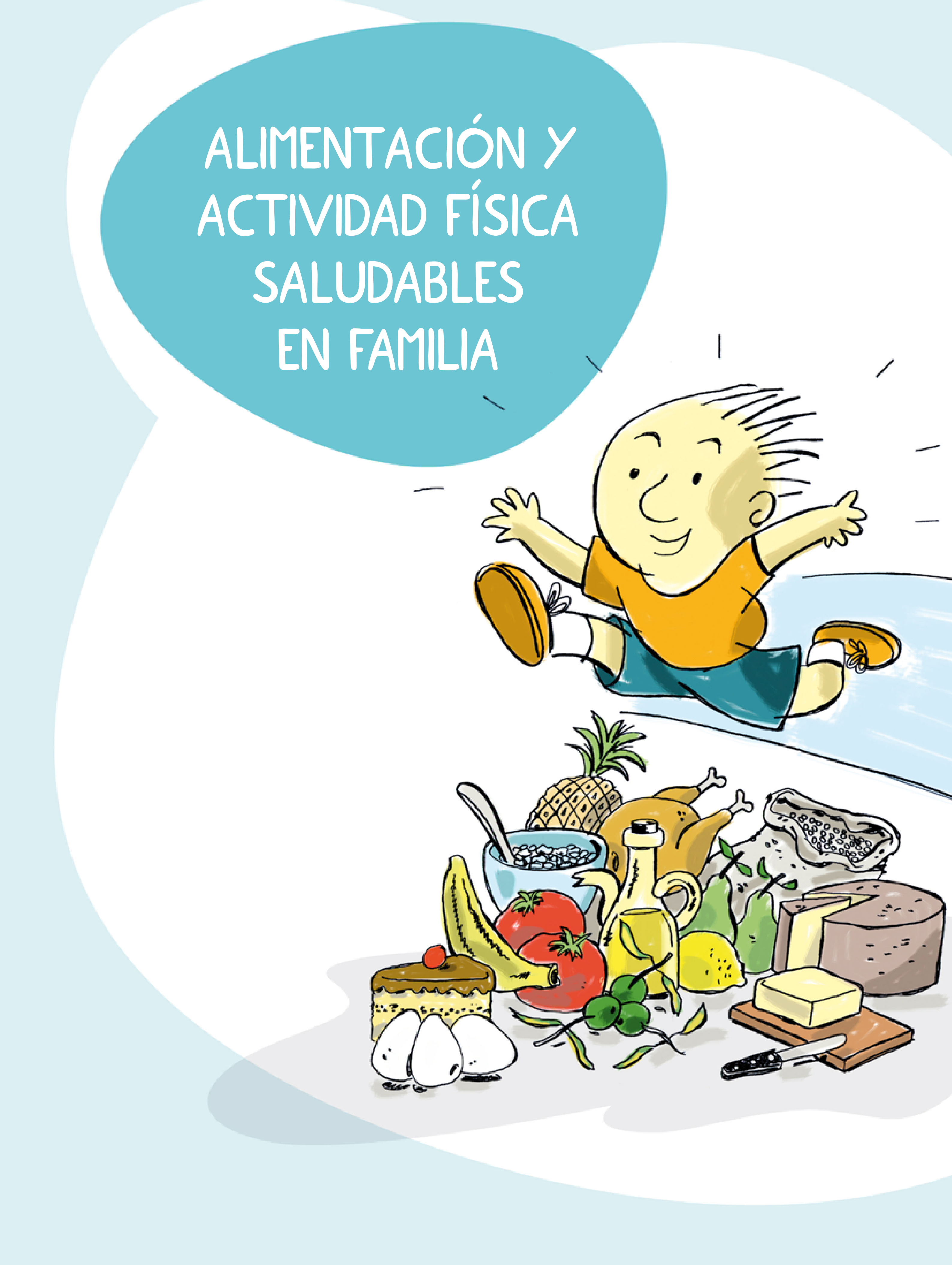

Supplement: Supplementary file 1 [file ijerph-18-01622-s001.zip › Document S1.pdf]
