# Supplementary material for: A Multimodal Intervention for Prevention of Overweight and Obesity in Schoolchildren. A Protocol Study “PREVIENE-CÁDIZ”
Source: Int J Environ Res Public Health. 2021 Feb 8;18(4):1622. doi: 10.3390/ijerph18041622 (PMC7914756; doi:10.3390/ijerph18041622)
Supplement: Supplementary file 1 [file ijerph-18-01622-s001.zip › Document S2.pdf]

Como comemos

¡Hola!

Desde la **Universidad de Cádiz y la Escuela Andaluza de Salud Pública estamos estudiando la alimentación de los niños y las niñas de tu edad en la provincia de Cádiz**. Te vamos a preguntar sobre lo que comes, la actividad física que haces y cómo te sientes.

No se trata de un examen, no hay respuestas buenas ni malas. Sólo tienes que dar tu opinión y contestar sobre lo que haces. Como **es anónimo**, nadie va a saber cuáles son tus respuestas.

¿Qué tienes que hacer?

- ✓ Lee atentamente cada pregunta y pregúntanos si no la entiendes bien.
- ✓ Responde la verdad, es importante.
- ✓ Si te equivocas puedes cambiar la respuesta. ¿Cómo? Táchala y marca la nueva.

Son 64 preguntas en esta encuesta.
¡Muchas gracias por participar!

Código: Antes de empezar, **por favor escribe el código que te han dado** para ésta encuesta. Recuerda que tendrás que utilizarlo otra vez, para la segunda parte.

Por favor, escribe **aquí**: **Código:**

Datos personales

Para empezar, queremos saber algunas cosas sobre ti.

1. Por favor, elige el año en que naciste.

- ○ 2008
- ○ 2009
- ○ 2010
- ○ 2011

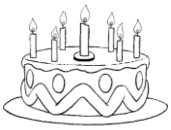

2. ¿Eres un chico o una chica?

- ○ Chico
- ○ Chica

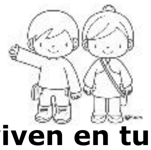

3. ¿Cuántas personas viven en tu casa? Incluyéndote a ti.

- ○ 2
- ○ 3
- ○ 4
- ○ 5
- ○ 6
- ○ 7
- ○ 8
- ○ 9
- ○ 10
- ○ Más de 10

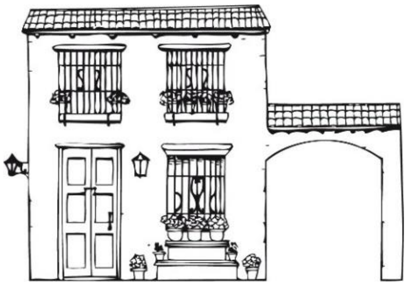

Datos familiares

4. ¿Con quién vives en tu casa? Señala todas las personas que viven contigo.
Por favor, marque *las opciones* que correspondan:

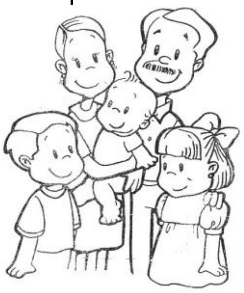

- ☐ Madre
- ☐ Padre
- ☐ Pareja de la madre
- ☐ Pareja del padre
- ☐ Abuela
- ☐ Abuelo
- ☐ Hermano/s
- ☐ Hermana/s
- ☐ Familia de acogida
- ☐ Centro o residencia de acogida
- ☐ Otra/s persona/s: ________

5. En tu casa, ¿Tienes un dormitorio solo para ti? Por favor, seleccione **sólo una** de las siguientes opciones:

- ○ Sí
- ○ No

6. ¿Cuántos ordenadores tenéis en tu familia? Por favor, seleccione **sólo una** de las siguientes opciones:

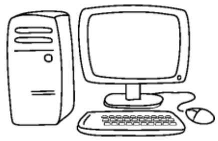

- ○ Ninguno
- ○ Uno
- ○ Dos
- ○ Más de dos

7. ¿Tiene tu familia coche propio o furgoneta? Por favor. seleccione **sólo una** de las siguientes opciones:

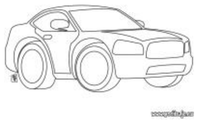

- ○ No
- ○ Sí, uno
- ○ Sí, dos o más.

8. Durante los últimos 12 meses, ¿Cuántas veces saliste de vacaciones con tu familia?
Por favor, seleccione **sólo una** de las siguientes opciones:

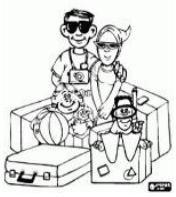

- ○ Nunca
- ○ Una vez
- ○ Dos veces
- ○ Más de dos veces

Comidas diarias

Ahora te vamos a preguntar sobre lo que comes.

9. ¿Qué comidas haces todos los días? Por favor, marca *las opciones* que correspondan:

- ☐ Desayuno (antes de empezar las clases)
- ☐ Desayuno a media mañana. Recreo
- ☐ Comida del mediodía
- ☐ Merienda
- ☐ Cena
- ☐ Antes de acostarte

10. Cuando te saltas el desayuno, ¿Te cuesta atender en la clase? Por favor, marca sólo **una**:

- ○ Sí
- ○ No

11. ¿Con quién sueles desayunar normalmente? Por favor, marque *las opciones* que correspondan:

☐ Madre
☐ Pareja de la madre
☐ Padre
☐ Pareja del padre
☐ Hermanos/as
☐ Abuelo/a
☐ Compañeros de escuela o de comedor escolar
☐ Amigos
☐ Nadie
☐ Otro: ____________

12. ¿Con quién sueles comer a mediodía normalmente? Por favor, marque *las opciones* que correspondan:

☐ Madre
☐ Pareja de la madre
☐ Padre
☐ Pareja del padre
☐ Hermanos/as
☐ Abuelo/a
☐ Compañeros de escuela o de comedor escolar
☐ Amigos
☐ Nadie
☐ Otro: ____________

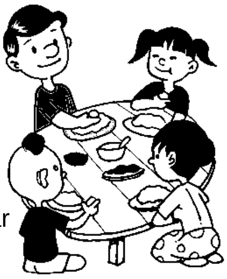

13. ¿Con quién sueles merendar normalmente? Por favor, marque *las opciones* que correspondan:

☐ Madre
☐ Pareja de la madre
☐ Padre
☐ Pareja del padre
☐ Hermanos/as
☐ Abuelo/a
☐ Compañeros de escuela o de comedor escolar
☐ Amigos
☐ Nadie
☐ Otro: ____________

14. ¿Con quién sueles cenar normalmente? Por favor, marque *las opciones* que correspondan:

☐ Madre
☐ Pareja de la madre
☐ Padre
☐ Pareja del padre
☐ Hermanos/as
☐ Abuelo/a
☐ Compañeros de escuela o de comedor escolar
☐ Amigos
☐ Nadie
☐ Otro: ____________

15. ¿Cenas viendo la televisión o de una pantalla (móvil, ordenador, Nintendo®, PlayStation®, Xbox®, GameBoy®, etc.)? Por favor, seleccione **sólo una** de las siguientes opciones:

○ Siempre o casi siempre
○ Muchas veces (3 o más veces a la semana)
○ Pocas veces (menos de 3 veces a la semana)
○ Nunca o casi nunca

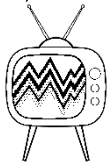
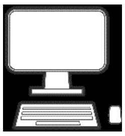

16. ¿Sueles beber agua mientras comes y cenas? Por favor, marca sólo **una** respuesta:

○ Siempre o casi siempre
○ Muchas veces (3 o más veces a la semana)
○ Pocas veces (menos de 3 veces a la semana)
○ Nunca o casi nunca

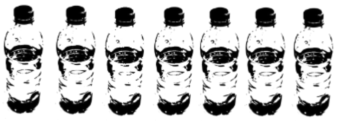

Desayuno

17. ¿Desayunas sentado o sentada en la mesa? Por favor, marca sólo **una** respuesta:

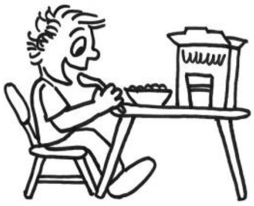

- ○ Siempre o casi siempre
- ○ Muchas veces (3 o más veces a la semana)
- ○ Pocas veces (menos de 3 veces a la semana)
- ○ Nunca o casi nunca

18. Todos o casi todos los días, ¿Desayunas antes de ir a la escuela? Por favor, marca sólo **una** respuesta:

- ○ Sí
- ○ No

19. Señala todos los alimentos que sueles desayunar, aunque no comas todos cada día.
Por favor, *marca las opciones* que correspondan:

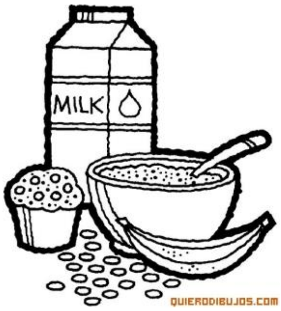

- ☐ Leche sola
- ☐ Leche con cacao, descafeinado, café, batidos
- ☐ Yogur, yogur líquido, Actimel®
- ☐ Pan, bocadillo, tostada
- ☐ Pan de molde (Bimbo®, pan árabe, tortitas)
- ☐ Cereales de desayuno
- ☐ Bollería o Pastas (galletas, croissants, suizos, donuts®, magdalenas, ensaimadas)
- ☐ Fruta (manzana, pera, naranja, mandarina, plátano etc.)
- ☐ Zumo
- ☐ Refrescos (Coca-cola®, Fanta®, Seven Up®, Sunny Delight®, Nestea®, etc.)
- ☐ Otros alimentos, indica cuáles:

20. ¿Qué alimentos sueles llevar para el recreo la mayoría de los días? Por favor, marca *las opciones* que correspondan:

- ☐ Leche sola
- ☐ Leche con cacao, descafeinado, café, batidos
- ☐ Yogur, yogur líquido, Actimel®
- ☐ Pan, bocadillo, tostada
- ☐ Pan de molde (Bimbo®, pan árabe, tortitas)
- ☐ Cereales de desayuno
- ☐ Bollería o pastas (galletas, croissants, suizos, donuts®, magdalenas, ensaimadas)
- ☐ Fruta (manzana, pera, naranja, mandarina, plátano etc.)
- ☐ Zumo
- ☐ Refrescos (Coca-cola®, Fanta®, Seven Up®, Sunny Delight®, Nestea®, etc.)
- ☐ Otros alimentos, indica cuáles:

Alimentos que comes y que te gustan

Ahora te vamos a preguntar por diferentes alimentos y te pedimos que nos digas *cuándo comes cada uno y si te gustan algunos de ellos*. No importa si no te acuerdas bien.

21. ¿Cuándo comes pan (barra, tostada, bocadillo)? Por favor, marca sólo **una** de las siguientes opciones:

- ○ Todos los días
- ○ Muchas veces (3 o 4 veces a la semana)
- ○ Pocas veces (1 o 2 veces a la semana)
- ○ Casi nunca (alguna vez al mes)
- ○ Nunca

22. ¿Cuándo comes pan de molde (pan Bimbo ®, pan de pita, tortitas, picos etc.)? Por favor, marca sólo **una** de las siguientes opciones:

- ○ Todos los días
- ○ Muchas veces (3 o 4 veces a la semana)
- ○ Pocas veces (1 o 2 veces a la semana)
- ○ Casi nunca (alguna vez al mes)
- ○ Nunca

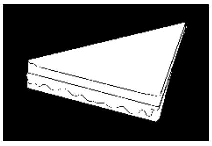

23. ¿Cuándo comes cereales de desayuno? Por favor, marca solo **una**:

- ○ Todos los días
- ○ Muchas veces (3 o 4 veces a la semana)
- ○ Pocas veces (1 o 2 veces a la semana)
- ○ Casi nunca (alguna vez al mes)
- ○ Nunca

24. ¿Cuándo comes patatas (<u>sin contar las patatas de bolsa o fritas</u>)? Por favor, marca sólo **una**:

- ○ Todos los días
- ○ Muchas veces (3 o 4 veces a la semana)
- ○ Pocas veces (1 o 2 veces a la semana)
- ○ Casi nunca (alguna vez al mes)
- ○ Nunca

25. ¿Cuándo comes arroz o pasta (macarrones, espaguetis, fideos, cuscús, etc.)? Por favor, marca sólo **una**:

- ○ Todos los días
- ○ Muchas veces (3 o 4 veces a la semana)
- ○ Pocas veces (1 o 2 veces a la semana)
- ○ Casi nunca (alguna vez al mes)
- ○ Nunca

26. ¿Cuándo comes verduras crudas o ensalada (lechuga, tomate, zanahoria, pepino, etc.)? Por favor, marca sólo **una**:

- ○ Todos los días
- ○ Muchas veces (3 o 4 veces a la semana)
- ○ Pocas veces (1 o 2 veces a la semana)
- ○ Casi nunca (alguna vez al mes)
- ○ Nunca

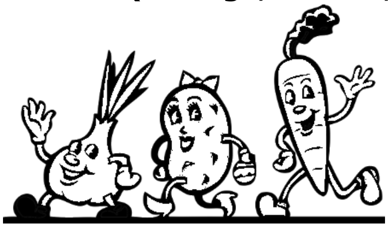

27. ¿Cuántas veces al día comes verduras crudas o ensalada? Por favor, marca sólo **una**:

- ○ Una vez al día
- ○ Dos veces al día
- ○ Más de dos veces al día

28. ¿Te gustan las verduras crudas o la ensalada (lechuga, tomate, zanahoria, pepino, etc.)? Por favor, selecciona sólo **una** respuesta:

- ○ Si
- ○ No

29. ¿Cuándo comes verduras cocinadas (espinacas, berenjenas, calabacín, judías verdes, etc.)? Por favor, marca sólo **una**:

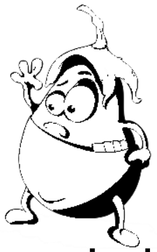

- ○ Todos los días
- ○ Muchas veces (3 o 4 veces a la semana)
- ○ Pocas veces (1 o 2 veces a la semana)
- ○ Casi nunca (alguna vez al mes)
- ○ Nunca

30. ¿Cuántas veces al día comes verduras cocinadas? Por favor, marca sólo **una**:

- ○ Una vez al día
- ○ Dos veces al día
- ○ Más de dos veces al día

31. ¿Te gustan algunas verduras cocinadas (espinacas, berenjena, calabacín, etc.)? Por favor marca sólo **una**:

- ○ Si
- ○ No

32. ¿Cuándo comes fruta (manzana, pera, naranja, plátano, etc.)? Por favor, marca sólo **una**:

- ○ Todos los días
- ○ Muchas veces (3 o 4 veces a la semana)
- ○ Pocas veces (1 o 2 veces a la semana)
- ○ Casi nunca (alguna vez al mes)
- ○ Nunca

33. ¿Cuántas veces al día comes fruta? Por favor, marca sólo **una**:

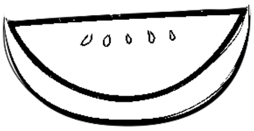

- ○ Una vez al día
- ○ Dos veces al día
- ○ Más de dos veces al día

34. ¿Te gusta la fruta? Por favor, marca sólo **una** de las siguientes opciones:

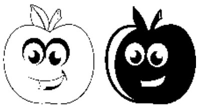

- ○ Si
- ○ No

35. ¿Cuándo bebes leche sola? Selecciona **una** de las siguientes opciones:

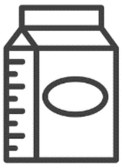

- ○ Todos los días
- ○ Muchas veces (3 o 4 veces a la semana)
- ○ Pocas veces (1 o 2 veces a la semana)
- ○ Casi nunca (alguna vez al mes)
- ○ Nunca

36. ¿Cuándo bebes leche con cacao, batidos, descafeinado? Por favor, marca sólo **una**:

- ○ Todos los días
- ○ Muchas veces (3 o 4 veces a la semana)
- ○ Pocas veces (1 o 2 veces a la semana)
- ○ Casi nunca (alguna vez al mes)
- ○ Nunca

37. ¿Cuándo comes yogurt, yogurt líquido o Actimel®? Por favor, marca sólo **una** de las siguientes opciones:

- ○ Todos los días
- ○ Muchas veces (3 o 4 veces a la semana)
- ○ Pocas veces (1 o 2 veces a la semana)
- ○ Casi nunca (alguna vez al mes)
- ○ Nunca

38. ¿Cuándo comes queso? Por favor, marca sólo **una** de las siguientes opciones:

- ○ Todos los días
- ○ Muchas veces (3 o 4 veces a la semana)
- ○ Pocas veces (1 o 2 veces a la semana)
- ○ Casi nunca (alguna vez al mes)
- ○ Nunca

39. ¿Cuándo comes pescado (merluza, sardinas, etc.)? Por favor, marca sólo **una**:

- ○ Todos los días
- ○ Muchas veces (3 o 4 veces a la semana)
- ○ Pocas veces (1 o 2 veces a la semana)
- ○ Casi nunca (alguna vez al mes)
- ○ Nunca

40. ¿Cuándo comes carne roja (ternera, cerdo, cordero, hamburguesas…)?
Por favor, marca sólo **una** respuesta:

- ○ Todos los días
- ○ Muchas veces (3 o 4 veces a la semana)
- ○ Pocas veces (1 o 2 veces a la semana)
- ○ Casi nunca (alguna vez al mes)
- ○ Nunca

41. ¿Cuándo comes carne blanca (pollo, pavo, conejo)? Por favor, marca sólo **una**:

- ○ Todos los días
- ○ Muchas veces (3 o 4 veces a la semana)
- ○ Pocas veces (1 o 2 veces a la semana)
- ○ Casi nunca (alguna vez al mes)
- ○ Nunca

42. ¿Cuándo comes huevos? Por favor, selecciona sólo **una**:

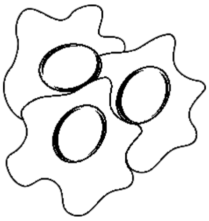

- ○ Todos los días
- ○ Muchas veces (3 o 4 veces a la semana)
- ○ Pocas veces (1 o 2 veces a la semana)
- ○ Casi nunca (alguna vez al mes)
- ○ Nunca

43. ¿Cuándo comes legumbres (judías o alubias, garbanzos, lentejas, guisantes, habas)?
Por favor, marca sólo **una** de las siguientes opciones:

- ○ Todos los días
- ○ Muchas veces (3 o 4 veces a la semana)
- ○ Pocas veces (1 o 2 veces a la semana)
- ○ Casi nunca (alguna vez al mes)
- ○ Nunca

44. ¿Cuándo comes frutos secos (almendras, avellanas, nueces, pistachos, cacahuetes, etc.)?
Por favor, marca sólo **una**:

- ○ Todos los días
- ○ Muchas veces (3 o 4 veces a la semana)
- ○ Pocas veces (1 o 2 veces a la semana)
- ○ Casi nunca (alguna vez al mes)
- ○ Nunca

45. ¿Cuándo comes postres lácteos (flan, natillas, arroz con leche, helados, etc.)?
Selecciona sólo **una**:

- ○ Todos los días
- ○ Muchas veces (3 o 4 veces a la semana)
- ○ Pocas veces (1 o 2 veces a la semana)
- ○ Casi nunca (alguna vez al mes)
- ○ Nunca

46. ¿Cuándo comes bollería o dulces (galletas, croissants, magdalenas, donuts ®)?
Por favor, marca sólo **una**:

- ○ Todos los días
- ○ Muchas veces (3 o 4 veces a la semana)
- ○ Pocas veces (1 o 2 veces a la semana)
- ○ Casi nunca (alguna vez al mes)
- ○ Nunca

47. ¿Cuándo comes chucherías dulces (caramelos, chicles, gominolas, chocolatinas, etc.)?
Por favor, marca sólo **una** de las siguientes opciones:

- ○ Todos los días
- ○ Muchas veces (3 o 4 veces a la semana)
- ○ Pocas veces (1 o 2 veces a la semana)
- ○ Casi nunca (alguna vez al mes)
- ○ Nunca

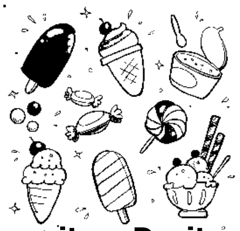

48. ¿Cuándo comes patatas de bolsa, gusanitos, Doritos®, etc.? Por favor, marca sólo **una**:

- ○ Todos los días
- ○ Muchas veces (3 o 4 veces a la semana)
- ○ Pocas veces (1 o 2 veces a la semana)
- ○ Casi nunca (alguna vez al mes)
- ○ Nunca

49. ¿Cuándo comes embutido (jamón serrano, salchichón, chorizo, salchicha, mortadela, beicon, etc.)? Por favor, marca sólo **una** de las siguientes opciones:

- ○ Todos los días
- ○ Muchas veces (3 o 4 veces a la semana)
- ○ Pocas veces (1 o 2 veces a la semana)
- ○ Casi nunca (alguna vez al mes)
- ○ Nunca

50. ¿Cuándo bebes zumo de fruta envasado? Por favor, selecciona sólo **una** de las siguientes opciones:

- ○ Todos los días
- ○ Muchas veces (3 o 4 veces a la semana)
- ○ Pocas veces (1 o 2 veces a la semana)
- ○ Casi nunca (alguna vez al mes)
- ○ Nunca

51. ¿Cuándo bebes refrescos (Fanta®, Sunny Deliht®, Coca-Cola®, Nestea®, Aquarius®, Seven Up®)? Por favor, marca sólo **una** de las siguientes opciones:

- ○ Todos los días
- ○ Muchas veces (3 o 4 veces a la semana)
- ○ Pocas veces (1 o 2 veces a la semana)
- ○ Casi nunca (alguna vez al mes)
- ○ Nunca

Comer fuera de casa

52. Si comes en el comedor ¿Te acabas la comida que sirven en la escuela? Por favor, marca sólo **una** de las siguientes opciones:

- ○ Siempre
- ○ Muchas veces
- ○ Pocas veces
- ○ Nunca

53. Si comes en el comedor, ¿Te gusta la comida que sirven en la escuela? Por favor, seleccione sólo **una** de las siguientes opciones:

- ○ Si
- ○ No

54. ¿Cuántas veces vas a un establecimiento de comida rápida? (McDonalds®, Burger King®, Kentucky®, Telepizza®, Doner Kebab, Frankfurt, etc.). Por favor, marca sólo **una** de las siguientes opciones:

- ○ Todos los días
- ○ Muchas veces (3 o más veces a la semana)
- ○ Pocas veces (menos de 3 veces a la semana)
- ○ Nunca

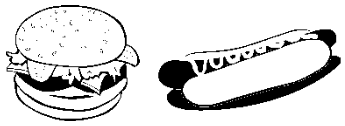

55. ¿Cuántas veces vas a comer a un restaurante? *No tengas en cuenta los establecimientos de comida rápida de la pregunta anterior*. Elige **una** respuesta**.**

- ○ Todos los días
- ○ Muchas veces (3 o más veces a la semana)
- ○ Pocas veces (menos de 3 veces a la semana)
- ○ Nunca

56. ¿Te gusta probar nuevos alimentos? Por favor, seleccione sólo **una** de las siguientes opciones:

- ○ Si
- ○ No

¿Qué piensas sobre los alimentos?

Las preguntas que hacemos ahora *son sobre lo que sientes y piensas*, aunque no sea lo que haces siempre.

57. De todos estos grupos de alimentos, ¿De cuáles deberías comer MÁS raciones cada día?
Por favor, selecciona sólo **una** de las siguientes opciones:

- ○ Pan, cereales, arroz, pasta
- ○ Productos lácteos (leche, yogur, queso)
- ○ Bollería (galletas, croissants, etc.), Golosinas
- ○ Fruta
- ○ Carne, pescado, huevos, frutos secos
- ○ Verduras
- ○ No lo sé

58. De todos estos grupos de alimentos, ¿De cuáles deberías comer MENOS raciones cada día? Marca sólo **una**:

- ○ Pan, cereales, arroz, pasta
- ○ Productos lácteos (leche, yogur, queso)
- ○ Bollería (galletas, croissants, etc.), Golosinas
- ○ Fruta
- ○ Carne, pescado, huevos, frutos secos
- ○ Verduras
- ○ No lo sé

59. ¿Cuántas veces al día <u>deberías</u> comer fruta y verduras? Por favor, marca sólo **una**:

- ○ Ninguna vez
- ○ 1 vez
- ○ 2 veces
- ○ 3 veces
- ○ 4 veces
- ○ 5 veces
- ○ No lo sé

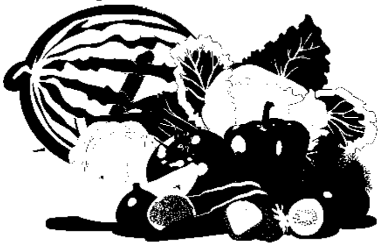

60. Lo que comes ¿Puede hacer que tengas una enfermedad cuando seas mayor? (del corazón, cáncer, etc.) Por favor, marca sólo **una**:

- ○ Sí
- ○ No
- ○ No lo sé

61. La gente que pesa más de lo que debería ¿Puede tener más problemas de salud que otras personas? Por favor, marca sólo **una**:

- ○ Sí
- ○ No
- ○ No lo sé

¿Qué opinas sobre la comida?

62. ¿Piensas que los alimentos que comes son buenos para tu salud? Por favor, marca sólo **una**:

- ○ Todos
- ○ Muchos
- ○ Pocos
- ○ Ninguno

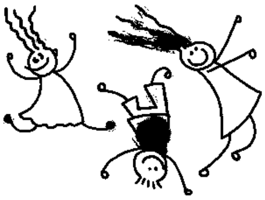

63. En nuestros barrios y pueblos hay recursos o actividades que nos dan bienestar y facilitan mantener una alimentación saludable.

Indica **un recurso** **de tu barrio o pueblo** que facilita mantener una alimentación saludable.

Situaciones especiales

64. Si tu alimentación es distinta a la de tus compañeros y compañeras por alguna de éstas razones, márcalas por favor. **Puedes elegir varias:**

- ☐ Soy vegetariano (no como carne ni pescado)
- ☐ No como carne de cerdo
- ☐ Tengo alergia al gluten (no puedo comer pan o pasta)
- ☐ Soy intolerante a la lactosa (no puedo beber leche)
- ☐ Tengo alergia a los frutos secos
- ☐ Otras. ¿Cuáles?:

¡Ya has terminado!

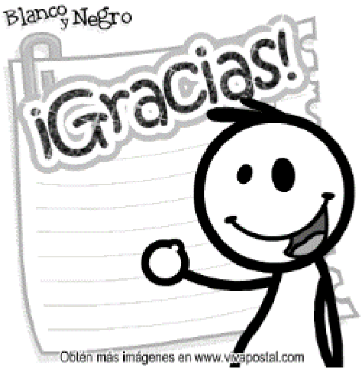

¡Saludos y gracias por completar esta encuesta!
