# Supplementary material for: A Multimodal Intervention for Prevention of Overweight and Obesity in Schoolchildren. A Protocol Study “PREVIENE-CÁDIZ”
Source: Int J Environ Res Public Health. 2021 Feb 8;18(4):1622. doi: 10.3390/ijerph18041622 (PMC7914756; doi:10.3390/ijerph18041622)

Como nos movemos y nos sentimos

¡Hola!

Desde la **Universidad de Cádiz y la Escuela Andaluza de Salud Pública estamos estudiando la alimentación de los niños y las niñas de tu edad en la provincia de Cádiz**. Te vamos a preguntar sobre la actividad física que haces y cómo te sientes.

No se trata de un examen, no hay respuestas buenas ni malas. Sólo tienes que dar tu opinión y contestar sobre lo que haces. Como **es anónimo**, nadie va a saber cuáles son tus respuestas.

¿Qué tienes que hacer?

- ✓ Lee atentamente cada pregunta y pregúntanos si no la entiendes bien.
- ✓ Responde la verdad, es importante.
- ✓ Si te equivocas puedes cambiar la respuesta. ¿Cómo? Táchala y marca la nueva.

Son 31 preguntas en esta encuesta.
¡**Muchas gracias por participar!**

CÓDIGO: Antes de empezar, **escribe el código que te han dado** para ésta encuesta**. Es el mismo que el utilizado en la encuesta de Alimentación**.

Por favor, escribe aquí: **Código:**

Actividad física

Te preguntamos sobre la actividad física que haces y algunos momentos de tu tiempo libre.

1. **Ahora nos gustaría que nos contestaras**. Por favor, selecciona la respuesta apropiada para cada concepto:

	Caminando	En bicicleta	En transporte público (bus)	En coche	Otros
**¿Cómo vas a la escuela?**	○	○	○	○	○
**¿Cómo vuelves de la escuela?**	○	○	○	○	○

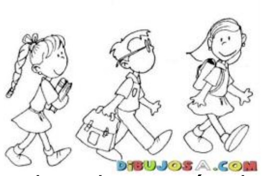

2. **¿Qué haces durante el recreo?** *Puedes elegir más de una respuesta.*

☐ Estoy sentado/a (hablando, leyendo, jugando a muñecas, jugando videojuegos)
☐ Juego en los columpios u otros juegos de moverse que hay en el patio de la escuela
☐ Juego a pelota, correr, cuerda, bailar, etc.
☐ Juego fútbol, baloncesto, etc.
☐ Otro:

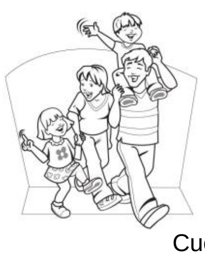

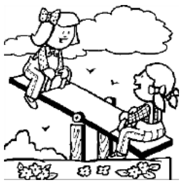

3. ¿Cuántos días a la semana tienes clase de Educación Física?
Por favor, marca ***sólo una*** *de las siguientes opciones:*

- ○ Ningún día
- ○ 1 día
- ○ 2 días
- ○ 3 días o más

4. Durante los días de semana y una vez terminadas las clases, ¿Participas en actividades deportivas <u>con entrenador o monitor</u>? (por ejemplo: natación, fútbol, danza, etc.).
Por favor, selecciona ***sólo una****:*

- ○ Sí
- ○ No

5. ¿En qué actividades deportivas <u>con entrenador o monitor</u> participas (<u>una vez terminadas las clases</u>)? (por ejemplo: natación, fútbol, danza, etc.).
Puedes elegir varias.

- ☐ Atletismo
- ☐ Baloncesto
- ☐ Danza
- ☐ Esquí
- ☐ Fútbol
- ☐ Gimnasia (rítmica, acrobática etc.)
- ☐ Natación
- ☐ Rugby
- ☐ Tenis
- ☐ Voleibol
- ☐ Otro: ____________________

6. ¿Cuántos días a la semana practicas esa actividad? (por ejemplo: natación, fútbol, danza, etc.). *Por favor, selecciona la respuesta apropiada para cada concepto:*

	Atletismo	Baloncesto	Danza	Esquí	Fútbol	Gimnasia	Natación	Rugby	Tenis	Voleibol	Otros
**1 día**	○	○	○	○	○	○	○	○	○	○	○
**2 días**	○	○	○	○	○	○	○	○	○	○	○
**3 días**	○	○	○	○	○	○	○	○	○	○	○
**4 o más días**	○	○	○	○	○	○	○	○	○	○	○

7. ¿Y podrías decir cuántas horas cada día practicas esa actividad? (por ejemplo: natación, fútbol, danza, etc.). *Por favor, selecciona la respuesta apropiada para cada concepto:*

	Atletismo	Baloncesto	Danza	Esquí	Fútbol	Gimnasia	Natación	Rugby	Tenis	Voleibol	Otros
**1 hora**	○	○	○	○	○	○	○	○	○	○	○
**1 hora y media**	○	○	○	○	○	○	○	○	○	○	○
**2 horas**	○	○	○	○	○	○	○	○	○	○	○
**2 horas y media**	○	○	○	○	○	○	○	○	○	○	○
**3 horas**	○	○	○	○	○	○	○	○	○	○	○
**3 horas y media**	○	○	○	○	○	○	○	○	○	○	○
**4 horas o más**	○	○	○	○	○	○	○	○	○	○	○

8. ¿Qué zonas hay cerca de tu casa para poder ir a jugar? *Puedes señalar más de una respuesta:*

- ☐ No hay ninguna
- ☐ Parque con juegos infantiles (columpios, etc.)
- ☐ Espacio amplio (plaza, parque, etc.) donde se puede ir en bicicleta, jugar a pelota, etc.
- ☐ Pabellón deportivo
- ☐ Otro: ____________________

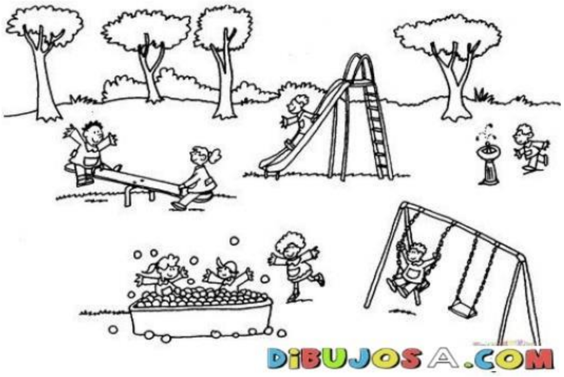

9. ¿Haces deporte, corres en bicicleta, patinas, vas a la montaña, etc., con tu familia? *Marca **una** respuesta.*

- ○ Sí
- ○ No

10. ¿Cuántos días de la semana haces deporte, montas en bicicleta, patinas, vas a la montaña, etc., con tu familia?
*Por favor, selecciona **sólo una**:*

○ 1 ○ 2 ○ 3 ○ 4 ○ 5 ○ 6 ○ 7

11. ¿Juegas o practicas algún deporte con tus amigos durante el fin de semana?
*Por favor, selecciona **sólo una**:*

- ○ Todos los fines de semanas del mes
- ○ Dos o tres fines de semana al mes
- ○ Un fin de semana al mes
- ○ Nunca

12. ¿Haces deporte o juegas con tu familia por las tardes durante la semana?
*Por favor, selecciona **sólo una** de las siguientes opciones:*

- ○ Todos los días de la semana
- ○ Dos o tres días a la semana
- ○ Un día a la semana
- ○ Nunca

13. En nuestros barrios y pueblos hay recursos o actividades que nos dan bienestar y facilitan realizar actividad física.

Indica **un recurso de tu barrio o pueblo** que facilite que realices actividad física.

____

TV y Videojuegos

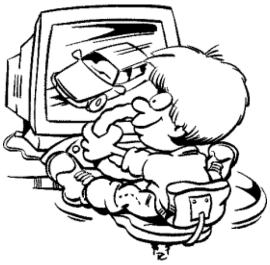

14. De lunes a jueves, sumando todo el tiempo que dedicas a estas actividades ¿cuántas horas al día ves la televisión, DVD, Nintendo®, PlayStation®, Xbox®, GameBoy®, Nintendo DS®, etc.? *Elige **una** respuesta.*

- ○ Ninguna vez
- ○ Menos de 2 horas al día
- ○ Entre 2 a 4 horas al día
- ○ Más de 4 horas al día

15.De viernes a domingo y festivos, ¿cuántas horas al día ves la televisión, DVD, Nintendo®, PlayStation®, Xbox®, GameBoy®, Nintendo DS®, etc.? *Elige **una** respuesta*

- ○ Ninguna vez
- ○ Menos de 2 horas al día
- ○ Entre 2 a 4 horas al día
- ○ Más de 4 horas al día

16. De lunes a jueves, ¿cuantas horas al día pasas delante de un ordenador o móvil navegando por internet, chateando con amigos, etc., una vez terminadas las clases? ***Marca una respuesta:***

- ○ Ninguna vez
- ○ Menos de 2 horas al día
- ○ Entre 2 a 4 horas al día
- ○ Más de 4 horas al día

17. De viernes a domingo y los festivos ¿cuántas horas al día pasas delante de un ordenador o móvil navegando por internet, chateando con amigos, etc., una vez terminadas las clases? ***Marca una respuesta:***

- ○ Ninguna vez
- ○ Menos de 2 horas al día
- ○ Entre 2 a 4 horas al día
- ○ Más de 4 horas al día

¿Cómo me siento?

Ahora te vamos a preguntar sobre ¿Cómo te sientes?

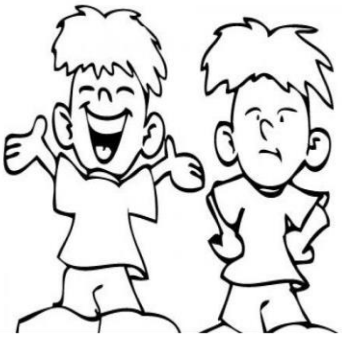

18. Dirías que tu salud es... *Elige **una** respuesta.*

- ○ Muy buena
- ○ Buena
- ○ Regular
- ○ Mala
- ○ No lo sé

19. En la última semana, ¿Te has sentido bien?
*Por favor, selecciona **sólo una** de las siguientes opciones:*

- ○ Nunca o casi nunca
- ○ Pocas veces (1 vez a la semana)
- ○ Algunas veces (2-3 veces a la semana)
- ○ Muchas veces (4-5 veces a la semana)
- ○ Siempre o casi siempre

20. ¿Eres feliz? *Por favor, selecciona **sólo una** de las siguientes opciones:*

- ○ Si
- ○ No

21. Durante la última semana, ¿Cuántas veces...
Por favor, selecciona la respuesta apropiada para cada concepto:

	¿Te ha costado respirar?	¿Has sentido picor en la piel?
**Nunca o casi nunca**	○	○
**Pocas veces (1 vez a la semana)**	○	○
**Algunas veces (2-3 veces a la semana)**	○	○
**Muchas veces (4-5 veces a la semana)**	○	○
**Siempre o casi siempre**	○	○

22. Durante la última semana, ¿Cuántas veces...
Por favor, selecciona la respuesta apropiada para cada concepto:

	¿Has llorado mucho?	¿Has estado preocupado o preocupada?	¿Has estado enfadado o enfadada?	¿Has sentido miedo?
**Nunca o casi nunca**	○	○	○	○
**Pocas veces (1 vez a la semana)**	○	○	○	○
**Algunas veces (2-3 veces a la semana)**	○	○	○	○
**Muchas veces (4-5 veces a la semana)**	○	○	○	○
**Siempre o casi siempre**	○	○	○	○

23. Durante la última semana, ¿Cuántas veces te has encontrado tan mal que no has tenido ganas de jugar en casa? *Marca **una** respuesta:*

- ○ Nunca o casi nunca
- ○ Pocas veces (1 vez a la semana)
- ○ Algunas veces (2-3 veces a la semana)
- ○ Muchas veces (4-5 veces a la semana)
- ○ Siempre o casi siempre

24. Durante la última semana, ¿Cuántas veces te has sentido tan mal que no has podido salir de casa? *Marca **una** respuesta:*

- ○ Nunca o casi nunca
- ○ Pocas veces (1 vez a la semana)
- ○ Algunas veces (2-3 veces a la semana)
- ○ Muchas veces (4-5 veces a la semana)
- ○ Siempre o casi siempre

25. ¿Estás contento o contenta con tu forma de ser? ***Por favor, marca sólo una respuesta:***

○ Si
○ No

26. En la última semana, ¿Cuántas veces te sentiste muy orgulloso de ti mismo/orgullosa de ti misma? *Marca* ***una*** *respuesta:*

○ Nunca o casi nunca
○ Pocas veces (1 vez a la semana)
○ Algunas veces (2-3 veces a la semana)
○ Muchas veces (4-5 veces a la semana)
○ Siempre o casi siempre

27. En la última semana, ¿Cuántas veces sentiste que te querían? *Marca* ***una*** *respuesta:*

○ Nunca o casi nunca
○ Pocas veces (1 vez a la semana)
○ Algunas veces (2-3 veces a la semana)
○ Muchas veces (4-5 veces a la semana)
○ Siempre o casi siempre

28. En la última semana, ¿Cuántas veces te lo pasaste muy bien? *Marca* ***una*** *respuesta:*

○ Nunca o casi nunca
○ Pocas veces (1 vez a la semana)
○ Algunas veces (2-3 veces a la semana)
○ Muchas veces (4-5 veces a la semana)
○ Siempre o casi siempre

29. En la última semana, ¿Cuántas veces te viste muy guapo o guapa? *Elige* ***una*** *respuesta*

○ Nunca o casi nunca
○ Pocas veces (1 vez a la semana)
○ Algunas veces (2-3 veces a la semana)
○ Muchas veces (4-5 veces a la semana)
○ Siempre o casi siempre

30. En la última semana, ¿Cuántas veces te sentiste sano o sana? *Marca* ***una*** *respuesta:*

○ Nunca o casi nunca
○ Pocas veces (1 vez a la semana)
○ Algunas veces (2-3 veces a la semana)
○ Muchas veces (4-5 veces a la semana)
○ Siempre o casi siempre

31. Cuando quiero algo, estoy seguro o segura que lo conseguiré. *Marca* ***sólo una*** *respuesta:*

○ Siempre
○ Frecuentemente
○ Algunas veces
○ Nunca
○ No sé/No contesto

¡Ya has terminado!

¡Gracias por completar esta encuesta!

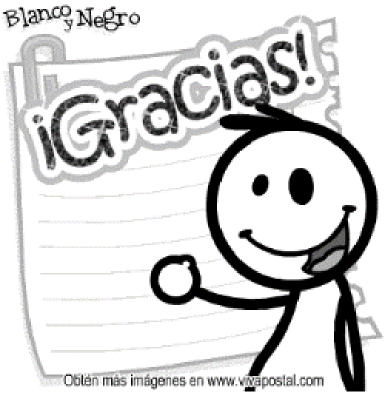

Supplement: Supplementary file 1 [file ijerph-18-01622-s001.zip › Document S3.pdf]
