# Supplementary material for: A Multimodal Intervention for Prevention of Overweight and Obesity in Schoolchildren. A Protocol Study “PREVIENE-CÁDIZ”
Source: Int J Environ Res Public Health. 2021 Feb 8;18(4):1622. doi: 10.3390/ijerph18041622 (PMC7914756; doi:10.3390/ijerph18041622)
Supplement: Supplementary file 1 [file ijerph-18-01622-s001.zip › Document S4.pdf]

Familias. Como comemos y nos movemos

¡Hola!

Desde la **Universidad de Cádiz y la Escuela Andaluza de Salud Pública** estamos estudiando los hábitos de alimentación, actividad física y horas de descanso de los niños, las niñas y sus familias en la provincia de Cádiz.

Para ello os pedimos vuestra colaboración. Se trata de un cuestionario para conocer estos hábitos. No le llevará más de 10/15 minutos y esta información es de una enorme importancia para nuestro estudio.

No se trata de un examen, no hay respuestas buenas ni malas. Sólo tiene que dar su opinión y contestar sobre lo que hace. Como es **anónimo**, nadie va a saber cuáles son sus respuestas.

¿Qué tiene que hacer?:

- Leer atentamente cada pregunta.
- Responder la verdad, es importante.
- Si se equivoca puede cambiar la respuesta. ¿Cómo? Táchela y marque la nueva.

¡Muchas gracias por participar!

Hay 55 preguntas en esta encuesta.

Antes de empezar, **por favor escriba el código que se le ha facilitado**:

Código:

Quién realiza el cuestionario

1. Por favor, indique quién realiza el cuestionario. Por favor, seleccione **sólo una** de las siguientes opciones:

- ○ Madre
- ○ Padre
- ○ Ambos
- ○ Otra persona:

Alimentación adultos

2. ¿Desayuna en las primeras horas de la mañana? Marque una respuesta. Por favor, seleccione **sólo una** de las siguientes opciones:

- ○ Nunca o casi nunca (menos de 1 vez a la semana)
- ○ Algunas veces (menos de 3 veces a la semana)
- ○ Muchas veces (de 3 a 5 veces a la semana)
- ○ Siempre o casi siempre (6 o 7 veces a la semana)

3. Si desayuna entre semana (de lunes a viernes) en las primeras horas de la mañana ¿Dónde lo hace más frecuentemente? Marque una respuesta. Por favor, seleccione **sólo una** de las siguientes opciones:

- ○ En casa
- ○ En el trabajo
- ○ En una cafetería
- ○ Otro lugar. Diga cuál:

4. ¿Toma el desayuno sentado/a? Marque una respuesta. Por favor, seleccione **sólo una** de las siguientes opciones:

- ○ Nunca o casi nunca (menos de 1 vez a la semana)
- ○ Algunas veces (menos de 3 veces a la semana)
- ○ Muchas veces (de 3 a 5 veces a la semana)
- ○ Siempre o casi siempre (6 o 7 veces a la semana)

5. Señale los alimentos que normalmente toma para desayunar (sin tener en cuenta los que tome a media mañana). Puede señalar más de una respuesta. Por favor, marque las opciones que correspondan:

- ☐ Leche sola
- ☐ Leche con cacao, café, batidos
- ☐ Yogurt o yogurt líquido o Actimel ®
- ☐ Pan (bocadillo, tostada)
- ☐ Cereales de desayuno
- ☐ Pastas (galletas, croissants, ensaimadas, donuts ®,magdalenas, etc)
- ☐ Pieza de fruta (manzana, pera, naranja, mandarinas, plátano, etc.)
- ☐ Zumo de fruta
- ☐ Refrescos (Coca-cola®, Pepsi®, Fanta ®, Seven up®, SunnyDeligth®, Nestea ®, etc)
- ☐ Otros alimentos, diga cuáles:

6. Señale lo que come frecuentemente a media mañana. Puede señalar más de una respuesta. Por favor, marque las opciones que correspondan:

- ☐ Nada
- ☐ Leche sola
- ☐ Leche con cacao, café, batidos
- ☐ Yogurt o yogurt líquido o Actimel ®
- ☐ Pan (bocadillo, tostada)
- ☐ Cereales de desayuno
- ☐ Pastas (galletas, croissants, ensaimadas, donuts ®,magdalenas, etc.)
- ☐ Pieza de fruta (manzana, pera, naranja, mandarinas, plátano, etc.)
- ☐ Zumo de fruta
- ☐ Refrescos (Coca-cola®, Pepsi®, Fanta ®, Seven up®, SunnyDeligth®, Nestea ®, etc.)
- ☐ Otros alimentos, diga cuáles:

7. ¿Qué bebe normalmente durante las comidas (almuerzo, cena etc.)? Puede señalar más de una **respuesta**. Por favor, marque **las opciones que correspondan**:

- ☐ Nada
- ☐ Agua
- ☐ Zumos de fruta
- ☐ Refrescos (Coca-cola®, Pepsi®, Fanta ®, Seven up®, SunnyDeligth®, Nestea ®, etc.)
- ☐ Vino, cerveza etc.
- ☐ Otros. Cuales:

8. A continuación le pedimos que nos conteste cual es la frecuencia con que consume los siguientes alimentos, señalando con una cruz en el espacio que corresponda:

	Menos de una vez al mes	1 vez al mes	2-3 veces al mes	1 día a la semana	2-4 días a la semana	5-6 días a la semana	1 vez al día	2 veces al día	Más de 2 veces al día
Pan (pan de barra, pan tostado, bocadillos)	○	○	○	○	○	○	○	○	○
Pan de molde	○	○	○	○	○	○	○	○	○
Cereales de desayuno	○	○	○	○	○	○	○	○	○
Patatas (todas menos las patatas de bolsa)	○	○	○	○	○	○	○	○	○
Arroz, pasta (macarrones, espagueti, pasta en sopas, cuscús)	○	○	○	○	○	○	○	○	○
Verduras crudas o ensalada (lechuga, tomate, zanahoria, pimiento, etc.)	○	○	○	○	○	○	○	○	○
Verduras cocinadas (espinacas, berenjenas, calabacín, judía tierna, etc.)	○	○	○	○	○	○	○	○	○
Fruta (manzana, pera, naranja, plátano, melón, papaya, dátiles, etc.)	○	○	○	○	○	○	○	○	○
Leche sola	○	○	○	○	○	○	○	○	○
Leche con cacao, café, batidos	○	○	○	○	○	○	○	○	○
Yogur, yogur líquido, Actimel ®	○	○	○	○	○	○	○	○	○
Queso	○	○	○	○	○	○	○	○	○
Legumbres (judías, lentejas, garbanzos, guisantes, habas, fríjoles, etc.)	○	○	○	○	○	○	○	○	○
Carne blanca (pollo, pavo, conejo)	○	○	○	○	○	○	○	○	○
Carne roja (hamburguesa, cerdo, ternera, cordero, etc.)	○	○	○	○	○	○	○	○	○
Pescado (merluza, bacalao, dorada, salmón, atún, sardina, boquerones)	○	○	○	○	○	○	○	○	○

	Menos de una vez al mes	1 vez al mes	2-3 veces al mes	1 día a la semana	2-4 días a la semana	5-6 días a la semana	1 vez al día	2 veces al día	Más de 2 veces al día
**Huevos (tortilla, huevo frito, huevo hervido, etc.)**	○	○	○	○	○	○	○	○	○
**Frutos secos (almendras, avellanas, nueces, etc.)**	○	○	○	○	○	○	○	○	○
**Postres lácteos (flan, natillas, crema catalana, helados, etc.)**	○	○	○	○	○	○	○	○	○
**Bollería (galletas, croissants, donuts ® , magdalenas)**	○	○	○	○	○	○	○	○	○
**Chucherías (caramelos, chicles, chocolate, etc.)**	○	○	○	○	○	○	○	○	○
**Patatas de bolsa, ganchitos, palomitas de bolsa, Bocabits ®, Doritos®**	○	○	○	○	○	○	○	○	○
**Embutidos (jamón serrano, butifarra, salami, mortadela, beicon, etc.)**	○	○	○	○	○	○	○	○	○
**Zumo de fruta**	○	○	○	○	○	○	○	○	○
**Agua**	○	○	○	○	○	○	○	○	○
**Refrescos (Coca-cola ®, Pepsi ®, Fanta ®, Seven up ®, SunnyDelight ®,Nestea®, etc.)**	○	○	○	○	○	○	○	○	○
**Vino, cerveza**	○	○	○	○	○	○	○	○	○
**Whisky, ginebra, vodka, ron, etc.**	○	○	○	○	○	○	○	○	○

Alimentación hijos/as

Ahora le pedimos que conteste a unas preguntas sobre **lo que comen sus hijos/as**.

9. ¿Su hijo/a desayuna antes de empezar las clases? (Tanto si desayuna en casa, como si lo hace en la escuela). Por favor, seleccione **sólo una** de las siguientes opciones:

- ○ Nunca o casi nunca (menos de 1 vez a la semana)
- ○ Algunas veces (menos de 3 veces a la semana)
- ○ Muchas veces (de 3 a 5 veces a la semana)
- ○ Siempre o casi siempre (6 o 7 veces a la semana)

10. ¿Su hijo o hija toma el desayuno sentado/a y en la mesa antes de empezar las clases? Por favor, seleccione **sólo una** de las siguientes opciones:

- ○ Nunca o casi nunca (menos de 1 vez a la semana)
- ○ Algunas veces (menos de 3 veces a la semana)
- ○ Muchas veces (de 3 a 5 veces a la semana)
- ○ Siempre o casi siempre (6 o 7 veces a la semana)

11. Señale los alimentos que él o ella normalmente come para desayunar. *Puede señalar más de una respuesta.* Por favor, marque **las opciones que correspondan**:

- ☐ Leche sola
- ☐ Leche con cacao, café, batidos
- ☐ Yogur o yogur líquido o Actimel ®
- ☐ Pan (bocadillo, tostada)
- ☐ Cereales de desayuno
- ☐ Pastas (galletas, croissants, ensaimadas, donuts ®, magdalenas, etc.)
- ☐ Pieza de fruta (manzana, pera, naranja, mandarinas, plátano, etc.)
- ☐ Zumo de fruta
- ☐ Pan de molde
- ☐ Refrescos (Coca-cola®, Pepsi®, Fanta ®, Seven up®, SunnyDeligth®, Nestea ®, etc.)
- ☐ Otros alimentos, diga cuáles: ____________________

12. Señale lo que él o ella come a media mañana. *Puede señalar más de una respuesta*. Por favor, marque **las opciones que correspondan**:

- ☐ Nada
- ☐ Leche sola
- ☐ Leche con cacao, café, batidos
- ☐ Yogur o yogur líquido o Actimel ®
- ☐ Pan (bocadillo, tostada)
- ☐ Pan de molde
- ☐ Cereales de desayuno
- ☐ Bollería (galletas, croissants, ensaimadas, donuts ®,magdalenas, etc.)
- ☐ Pieza de fruta (manzana, pera, naranja, mandarinas, plátano, etc.)
- ☐ Zumo de fruta
- ☐ Refrescos (Coca-cola®, Pepsi®, Fanta ®, Seven up®, SunnyDeligth®, Nestea ®, etc.)
- ☐ Otros alimentos, diga cuáles: ____________________

13. ¿Qué bebe su hijo o hija normalmente durante las comidas (almuerzo, cena etc.)? *Puede señalar más de una respuesta*. Por favor, marque **las opciones que correspondan**:

- ☐ Nada
- ☐ Agua
- ☐ Zumos de fruta
- ☐ Refrescos (Coca-cola®, Pepsi®, Fanta ®, Seven up®, SunnyDeligth®, Nestea ®, etc.)
- ☐ Otros. Cuales: ______

14. A continuación le pedimos que nos conteste cual es la frecuencia con que su hijo o hija consume los siguientes alimentos:

	Menos de una vez al mes	1 vez al mes	2-3 veces al mes	1 día a la semana	2-4 días a la semana	5-6 días a la semana	1 vez al día	2 veces al día	Más de 2 veces al día
Pan (pan de barra, pan tostado, bocadillos)	○	○	○	○	○	○	○	○	○
Pan de molde	○	○	○	○	○	○	○	○	○
Cereales de desayuno	○	○	○	○	○	○	○	○	○
Patatas (todas menos las patatas de bolsa)	○	○	○	○	○	○	○	○	○
Arroz, pasta (macarrones, espagueti, pasta en sopas, cuscús)	○	○	○	○	○	○	○	○	○
Verduras crudas o ensalada (lechuga, tomate, zanahoria, pimiento, etc.)	○	○	○	○	○	○	○	○	○
Verduras cocinadas (espinacas, berenjenas, calabacín, judía tierna, etc.)	○	○	○	○	○	○	○	○	○
Fruta (manzana, pera, naranja, plátano, melón, papaya, dátiles, etc.)	○	○	○	○	○	○	○	○	○
Leche sola	○	○	○	○	○	○	○	○	○
Leche con cacao, café, batidos	○	○	○	○	○	○	○	○	○
Yogur, yogur líquido, Actimel ®	○	○	○	○	○	○	○	○	○
Queso	○	○	○	○	○	○	○	○	○
Legumbres (judías, lentejas, garbanzos, guisantes, habas, fríjoles, etc.)	○	○	○	○	○	○	○	○	○
Carne blanca (pollo, pavo, conejo)	○	○	○	○	○	○	○	○	○

	Menos de una vez al mes	1 vez al mes	2-3 veces al mes	1 día a la semana	2-4 días a la semana	5-6 días a la semana	1 vez al día	2 veces al día	Más de 2 veces al día
**Carne roja (hamburguesa, cerdo, ternera, cordero, etc.)**									
**Pescado (merluza, bacalao, dorada, salmón, atún, sardina, boquerones)**									
**Huevos (tortilla, huevo frito, huevo hervido, etc.)**									
**Frutos secos (almendras, avellanas, nueces, etc.)**									
**Postres lácteos (flan, natillas, crema catalana, helados, etc.)**									
**Bollería (galletas, croissants, donuts ®, magdalenas)**									
**Chucherías (caramelos, chicles, chocolate, etc.)**									
**Patatas de bolsa, ganchitos, palomitas de bolsa, Bocabits ®, Doritos ®**									
**Embutidos (jamón serrano, butifarra, salami, mortadela, beicon, etc.)**									
**Zumo de fruta**									
**Agua**									
**Refrescos (Coca-cola ®, Pepsi ®, Fanta ®, Seven up ®, SunnyDelight ®, Nestea ®, etc.)**									
**Vino, cerveza**									
**Whisky, ginebra, vodka, ron, etc.**									

Hábitos familiares

En las siguientes cuestiones le preguntamos sobre la forma de cocinar en casa, y como se suelen hacer las comidas.

15. ¿Utilizan aceite de oliva en casa, como principal grasa? Por favor, seleccione **sólo una** de las siguientes opciones:

- ○ Sí
- ○ No

16. Normalmente ¿Su hijo o hija mira la televisión o está delante de alguna pantalla (ordenador, móvil, Nintendo®, PlayStation®, Xbox®, Gameboy ®, etc.) durante las comidas? Por favor, seleccione **sólo una** de las siguientes opciones:

- ○ Nunca o casi nunca (menos de 1 vez a la semana)
- ○ Algunas veces (menos de 3 veces a la semana)
- ○ Muchas veces (de 3 a 5 veces a la semana)
- ○ Siempre o casi siempre (6 o 7 veces a la semana)

17. ¿Con qué frecuencia su hijo o hija va a un establecimiento de comida rápida? (McDonals ®, Burger King ®, Kentucky ®, Telepizza ®, Doner Kebab, Frankfrut, etc.). Por favor, seleccione **sólo una** de las siguientes opciones:

- ○ Nunca o casi nunca (hasta una vez al mes)
- ○ Algunas veces (de 2 a 3 veces al mes a 1 día a la semana)
- ○ Muchas veces (de 2 a 4 días a la semana)
- ○ Siempre o casi siempre (más de 4 días a la semana)

18. ¿Con qué frecuencia su hijo o hija va a comer en un restaurante? No tenga en cuenta los establecimientos de comida rápida. Por favor, seleccione **sólo una** de las siguientes opciones:

- ○ Nunca o casi nunca (hasta una vez al mes)
- ○ Algunas veces (de 2 a 3 veces al mes a 1 día a la semana)
- ○ Muchas veces (de 2 a 4 días a la semana)
- ○ Siempre o casi siempre (más de 4 días a la semana)

19. ¿Dónde acostumbran a hacer la compra de alimentos preferentemente? Por favor, seleccione la respuesta apropiada para cada concepto:

	**Productos frescos**	**Productos NO Perecederos**
**Grandes Superficies (Carrefour ®, Alcampo ®, etc.)**	○	○
**Supermercado (Mercadona ®, Caprabo ®, etc.)**	○	○
**Mercado**	○	○
**Pequeño comercio (tiendas del barrio)**	○	○
**Otros**	○	○

Conocimientos sobre los alimentos

Conteste a las siguientes preguntas **según lo que usted cree o piensa**, independientemente de que lo haga o no:

20. De todos estos grupos de alimentos, ¿De qué grupo considera que su hijo o hija debería comer el MAYOR número de raciones cada día? Por favor, seleccione **sólo una** de las siguientes opciones:

- ◯ Pan, arroz, pasta
- ◯ Productos lácteos (leche, queso, yogurt)
- ◯ Bollería (galletas, croissants, etc.), golosinas
- ◯ Fruta
- ◯ Carne
- ◯ Pescado
- ◯ Huevos
- ◯ Frutos secos
- ◯ Verduras y hortalizas
- ◯ No lo sé

21. De todos estos grupos de alimentos, ¿De qué grupo considera que su hijo o hija debería comer el MENOR número de raciones cada día? Por favor, seleccione **sólo una** de las siguientes opciones:

- ◯ Pan, arroz, pasta
- ◯ Productos lácteos (leche, queso, yogurt)
- ◯ Bollería (galletas, croissants, etc.), golosinas
- ◯ Fruta
- ◯ Carne
- ◯ Pescado
- ◯ Huevos
- ◯ Frutos secos
- ◯ Verduras y hortalizas
- ◯ No lo sé

22. ¿Cuántas raciones en total de frutas, verduras y hortalizas debería comer su hijo o hija CADA DÍA? Por favor, seleccione **sólo una** de las siguientes opciones:

- ◯ 2 raciones
- ◯ 3 raciones
- ◯ 4 raciones
- ◯ 5 raciones como mínimo
- ◯ No lo sé

23. En nuestros barrios y pueblos hay recursos o actividades que nos dan bienestar y facilitan mantener una alimentación saludable.

Indique **un recurso de su barrio o pueblo** que facilite mantener una alimentación saludable para su familia.

24. Ahora le pedimos que conteste sobre lo que piensa acerca de la alimentación de SU HIJO O HIJA. ¿Hasta qué punto está de acuerdo con éstas afirmaciones?

	Totalmente en desacuerdo	Bastante en desacuerdo	Ni de acuerdo ni en desacuerdo	Bastante de acuerdo	Totalmente de acuerdo
Algunos alimentos deben estar fuera del alcance de los niños y niñas	○	○	○	○	○
Los niños y niñas deben comer muchos dulces (golosinas, caramelos, helados, pasteles o bollería)	○	○	○	○	○
Los padres y madres deben estar atentos a que sus hijos o hijas estén por encima del peso que les correspondería	○	○	○	○	○
Es importante que los niños y niñas coman los alimentos que les gustan cuando lo quieran	○	○	○	○	○
Los niños y niñas deben comer muchos alimentos como bollería, chuchería, patatas de bolsa, embutidos, etc.	○	○	○	○	○
Los padres y madres deben preocuparse si su hijo o hija tiene que hacer dieta para mantenerse en un peso deseable	○	○	○	○	○
Los zumos comerciales son saludables para sus hijos	○	○	○	○	○
Cuando los niños y niñas se portan bien, deben recompensarse con helados, bollería, caramelos, etc.	○	○	○	○	○
Los niños y niñas deben de terminarse la comida que tienen en el plato	○	○	○	○	○
Si no presto atención, mi hijo o hija come mucho de sus alimentos preferidos	○	○	○	○	○
Si no presto atención mi hijo o hija come mucho más de lo que debería	○	○	○	○	○
Cuando puedo, ofrezco a mi hijo o hija sus alimentos preferidos como recompensa por haberse portado bien conmigo	○	○	○	○	○
He de estar especialmente pendiente para asegurarme de que mi hijo o hija como lo suficiente	○	○	○	○	○
Si mi hijo o hija dice: "No tengo hambre", igualmente intento que coma	○	○	○	○	○
Creo que mi hijo o hija come adecuadamente	○	○	○	○	○

Actividad física

A continuación, le pedimos que conteste a unas preguntas sobre **la actividad física y el tiempo de descanso** de su hijo o hija:

Trayecto CASA - ESCUELA - CASA

25. ¿Cómo va su hijo o hija a la escuela normalmente? Por favor, seleccione la respuesta apropiada para cada concepto:

	Casa – Escuela	Escuela – Casa
Caminando	○	○
En transporte público (autobús, tren, etc.)	○	○
En coche o moto	○	○
En bicicleta	○	○
Otros	○	○

ENTORNO

26. ¿Qué zonas hay cerca de su casa para que su hijo o hija juegue? Puede señalar más de una respuesta. Por favor, marque las opciones que correspondan:

- ☐ Nada
- ☐ Parque infantil (columpios, etc.)
- ☐ Espacio amplio (plaza, parque etc.), donde se puede ir en bicicleta, jugar a la pelota etc.
- ☐ Pabellón o pista deportiva
- ☐ Otro: __________

DEPORTE Y JUEGO ACTIVO

27. ¿Su hijo o hija participa en actividades deportivas con entrenador o monitor una vez terminadas las clases? (por ejemplo: natación, fútbol, danza, baloncesto, etc.). Por favor, seleccione **sólo una** de las siguientes opciones:

- ○ Sí
- ○ No

28. En caso afirmativo, ¿En qué actividades deportivas con entrenador o monitor participa su hijo o hija, una vez terminadas las clases? (ejemplo: natación, fútbol, danza, etc.). Por favor, marque las opciones que correspondan:

	Número de veces por semana	Número de horas por actividad
Atletismo		
Baloncesto		
Fútbol		
Balonmano		
Danza, baile		
Judo		
Natación		
Rugby		
Tenis		
Vela		
Surf		
Voleibol		
Otros		

29. Durante la SEMANA PASADA, ¿Cuántos días su hijo o hija practicó otras actividades <u>SIN entrenador o monitor</u>, al menos una hora, en su tiempo libre? (por ejemplo: caminar rápido, ir en bicicleta, patinar, jugar en el parque, ir de excursión etc.) Por favor, seleccione **sólo una** de las siguientes opciones:

- ○ Ninguna vez
- ○ 1 día
- ○ 2 días
- ○ 3 días
- ○ 4 días
- ○ 5 o más días

30. ¿Normalmente salen en familia a hacer actividades como caminar, pasear, ir en bicicleta, ir al parque etc.? Por favor, seleccione **sólo una** de las siguientes opciones:

- ○ Sí
- ○ No

31. En caso afirmativo, ¿Cuántos días de la semana hacen deporte, montan en bicicleta, patinan o van a la montaña, <u>en familia</u>? Por favor, seleccione **sólo una** de las siguientes opciones:

- ○ 1 día
- ○ 2 días
- ○ 3 días
- ○ 4 días
- ○ 5 días
- ○ 6 días
- ○ 7 días

32. En general, ¿Piensa que su hijo o hija hace suficiente actividad física? Por favor, seleccione **sólo una** de las siguientes opciones:

- ○ No, en absoluto
- ○ No, probablemente no
- ○ Sí, probablemente si
- ○ Sí, seguro

33. De media, ¿Cuánto tiempo PIENSA que tendría que practicar actividad física un niño o niña en edad escolar, ya sea jugando o haciendo deporte? Por favor, seleccione **sólo una** de las siguientes opciones:

- ○ Nada de actividad física. Menos de 1 h a la semana
- ○ Poco tiempo. 1-2h a la semana
- ○ Algún tiempo. 3-4 h a la semana
- ○ Mucho tiempo. 5-6h a la semana

34. En nuestros barrios y pueblos hay recursos o actividades que nos dan bienestar y facilitan realizar actividad física. Indique **<u>un</u> recurso de su barrio o pueblo** que facilite <u>realizar actividad física para su familia</u>.

35. ¿Para usted es importante que su hijo o hija haga actividades extraescolares? Por favor, seleccione **sólo una** de las siguientes opciones:

- ○ Sí
- ○ No

36. <u>Numere por orden de importancia</u> las siguientes actividades extraescolares según su criterio: "1" la que elige primero y "4" la que elige en último lugar.

- ☐ Actividades como ludoteca, manualidades, cerámica, teatro, música, ajedrez etc.
- ☐ Actividades deportivas (fútbol, natación, danza, baloncesto etc.)
- ☐ Estudio de idiomas (inglés, francés, alemán, etc.)
- ☐ Repaso - estudio (academia, profesor de refuerzo en casa, etc.)

37. ¿Cuántas horas al día dedica su hijo o hija a hacer los deberes?

	Lunes a jueves	Viernes a domingo y festivos
Ninguna		
Hasta 30 min		
Entre 30 min y 1 hora		
De 1 a 2 horas		
Más de 2 horas		

38. De LUNES A JUEVES, ¿Cuántas horas al día, su hijo o hija… Por favor, seleccione la respuesta apropiada para cada concepto:

	Nunca o casi nunca	Algunas horas (Hasta 2 horas al día)	Muchas horas (Entre 2 y 4 horas al día)	Siempre o casi siempre (más de 4 horas al día)
¿Ve la televisión, DVD?	○	○	○	○
¿Juega a videojuegos (Nintendo ®, PlayStation ®, Xbox ®, Gameboy ®, Nintendo DS ®, etc.?	○	○	○	○
¿Está delante de un ordenador /móvil fuera del horario escolar (Internet, jugando, chateando?	○	○	○	○

39. De VIERNES A DOMINGO Y FESTIVOS, ¿Cuántas horas al día, su hijo o hija… Por favor, seleccione la respuesta apropiada para cada concepto:

	Nunca o casi nunca	Algunas horas (Hasta 2 horas al día)	Muchas horas (Entre 2 y 4 horas al día)	Siempre o casi siempre (más de 4 horas al día)
¿Ve la televisión, DVD?	○	○	○	○
¿Juega a videojuegos (Nintendo ®, PlayStation ®, Xbox ®, Gameboy ®, Nintendo DS ®, etc.?	○	○	○	○
¿Está delante de un ordenador /móvil fuera del horario escolar (Internet, jugando, chateando?	○	○	○	○

HORAS DE SUEÑO

40. En las últimas 4 semanas, ¿Cuántas veces su HIJO o HIJA… Por favor, seleccione la respuesta apropiada para cada concepto:

	Nunca	Algún día	Muchos días	Todos los días
… ha tenido dificultad para dormirse?	○	○	○	○
… se ha despertado varias veces mientras dormía?	○	○	○	○
… se ha despertado demasiado pronto?	○	○	○	○

41. ¿A qué hora se va a dormir su hijo o hija de lunes a jueves? ☐

42. ¿A qué hora se va a dormir su hijo o hija de viernes a domingo y festivos? ☐

43. ¿A qué hora se levanta su hijo o hija de lunes a jueves? ☐

44. ¿A qué hora se levanta su hijo o hija de viernes a domingo y festivos? ☐

Datos sociodemográficos

Para terminar, le pedimos algunos datos personales.

45. ¿Cuántas personas viven en su casa? **Sólo** se pueden introducir **números** en este campo. Por favor, escriba su respuesta:

☐ **Personas** - número

46. De ellos son:

☐ **Niñas** - número

☐ **Niños** - número

☐ **Adultos** - número

☐ **Mayores de 65 años** - número

47. Persona/s que contesta/n el cuestionario:

**Mujer** ☐	**Edad -** número ☐	**Mujer** ☐	**Edad -** número ☐
**Mujer** ☐	**Edad -** número ☐	**Mujer** ☐	**Edad -** número ☐
**Hombre** ☐	**Edad -** número ☐	**Hombre** ☐	**Edad -** número ☐
**Hombre** ☐	**Edad -** número ☐	**Hombre** ☐	**Edad -** número ☐

48. ¿Cuál es el lugar de nacimiento del padre y de la madre del niño o niña que participa en el proyecto? Por favor, seleccione la respuesta apropiada para cada concepto:

	Padre	Madre
Andalucía (España)	○	○
Otra comunidad autónoma (España)	○	○
Argentina	○	○
Alemania	○	○
Bolivia	○	○
China	○	○
Colombia	○	○
Ecuador	○	○
Francia	○	○
Marruecos	○	○
Reino Unido	○	○
Rumanía	○	○
Rusia	○	○
Otro país Europa	○	○
Otro país América del Sur	○	○
Otro país América del Norte	○	○
Otro país Asia	○	○
Otro país África	○	○

49. Para los padres y /o madres que no nacieron en España. ¿Cuál es su tiempo de residencia aquí? Por favor, seleccione la respuesta apropiada para cada concepto:

	Padre	Madre
Menos de 1 año	○	○
Entre 1 y 5 años	○	○
Entre 6 y 10 años	○	○
Más de 10 años	○	○

50. ¿Cuál es el nivel más alto de estudios que han finalizado? Por favor, seleccione la respuesta apropiada para cada concepto:

	Padre	Madre
No sabe leer o escribir	◯	◯
No ha estudiado pero sabe leer y escribir	◯	◯
Estudios primarios (hasta 5º EGB, ingreso)	◯	◯
EGB completa (8º) o similar (bachiller elemental)	◯	◯
ESO (Educación Secundaria Obligatoria)	◯	◯
Estudios de FPI	◯	◯
Estudios de FPII	◯	◯
Estudios Secundarios (BUP, B. Superior)	◯	◯
Estudios universitarios de grado medio (Diplomatura)	◯	◯
Estudios universitarios de grado superior (Licenciatura)	◯	◯
Otros	◯	◯

51. ¿Cuál es su situación laboral actual? Por favor, seleccione la respuesta apropiada para cada concepto:

	Padre	Madre
Trabaja	◯	◯
Está en paro y ha trabajado antes	◯	◯
Busca primer empleo	◯	◯
Jubilado/a (trabajó anteriormente)	◯	◯
No trabaja fuera de casa	◯	◯
Estudiante	◯	◯
Incapacidad/invalidez permanente	◯	◯
Otros. Especificar	◯	◯

52. ¿Cuál es la ocupación que desempeña en la actualidad o la última que ha desempeñado?

53. ¿Cuál es su situación laboral actual, o en la última ocupación que ha desempeñado?

	Padre	Madre
Trabajador/a por cuenta ajena	◯	◯
Trabajador/a por cuenta propia, autónomo/a	◯	◯
Empresario/a o empleador/a de 10 o más asalariados/as	◯	◯
Empresario/a o empleador/a de menos de 10 asalariados/as	◯	◯
Gerente de empresa de 10 o más asalariados/as	◯	◯
Gerente de empresa de menos de 10 asalariados/as	◯	◯

54. Con los ingresos de su hogar, ¿Cómo suele llegar usted o en su caso, usted y su familia en la actualidad a fin de mes? Por favor, seleccione **sólo una** de las siguientes opciones:

- ◯ Con mucha dificultad
- ◯ Con dificultad
- ◯ Con cierta/alguna dificultad
- ◯ Con cierta alguna facilidad
- ◯ Con facilidad
- ◯ Con mucha facilidad

55. Por favor, indique el intervalo en el que están comprendidos los <u>ingresos totales</u> <u>netos</u> de su hogar, sumando todas las fuentes (si existe más de una) y <u>deduciendo las retenciones</u> a cuenta por impuestos, cotizaciones sociales y otros pagos asimilados. Por favor, seleccione **sólo una** de las siguientes opciones:

- ◯ Hasta 300 €
- ◯ De 301 € a 499 €
- ◯ De 500 € a 999 €
- ◯ De 1.000 € a 1.499 €
- ◯ De 1.500 € a 1.999 €
- ◯ De 2.000 € a 2.499 €
- ◯ De 2.500 € a 2.999 €
- ◯ De 3.000 € a 4.999 €
- ◯ Más de 5.000 €
- ◯ No sabe, no está seguro/a

¡Muchas gracias por completar esta encuesta!
