# Supplementary material for: A Multimodal Intervention for Prevention of Overweight and Obesity in Schoolchildren. A Protocol Study “PREVIENE-CÁDIZ”
Source: Int J Environ Res Public Health. 2021 Feb 8;18(4):1622. doi: 10.3390/ijerph18041622 (PMC7914756; doi:10.3390/ijerph18041622)
Supplement: Supplementary file 1 [file ijerph-18-01622-s001.zip › Document S5.pdf]

IFiS

INTERNATIONAL FITNESS SCALE

It is important that you do this test by yourself without taking into account the answers or opinion from other persons. Your answer is only useful for the progress of science and medicine. Please answer all the questions and do not leave any blank. Mark only one answer per question, and more important: be sincere. Thank you for your cooperation.

NAME: ____ DATE (dd-mm-yy): _____________

Please, think about your current level of physical fitness (compared with your friends) and choose the most appropriate answer.

My general physical fitness is:

Very poor (1) □

Poor (2) □

Average (3) □

Good (4) □

Very good (5) □

My cardiorespiratory fitness (capacity to do exercise, for instance long running) **is:**

Very poor (1) □

Poor (2) □

Average (3) □

Good (4) □

Very good (5) □

My muscular strength is:

Very poor (1) □

Poor (2) □

Average (3) □

Good (4) □

Very good (5) □

My speed / agility is:

Very poor (1) □

Poor (2) □

Average (3) □

Good (4) □

Very good (5) □

My flexibility is:

Very poor (1) □

Poor (2) □

Average (3) □

Good (4) □

Very good (5) □

IFIS has been developed by the PROFITH research group, Granada, Spain. Versions of IFIS in different languages and for different age groups are available at: http://profith.ugr.es/IFIS IFIS was originally design and validated under the umbrella of the HELENA study, original reference: Ortega et al. The International Fitness Scale (IFIS): usefulness of self-reported fitness in youth. Int J Epidemiol 2011;40:701-1. IFIS has also been validated in adults: Ortega et al. Scand J Med Sci Sports, 2013;23:749-57; in children: Sanchez-Lopez et al. Scand J Med Sci Sports. 2015;25:543-51;and in women with fibromyalgia: Alvarez-Gallardo et al. Arch Phys Med Rehabil. 2016;97:395-404.
