# Supplementary material for: A Multimodal Intervention for Prevention of Overweight and Obesity in Schoolchildren. A Protocol Study “PREVIENE-CÁDIZ”
Source: Int J Environ Res Public Health. 2021 Feb 8;18(4):1622. doi: 10.3390/ijerph18041622 (PMC7914756; doi:10.3390/ijerph18041622)
Supplement: Supplementary file 1 [file ijerph-18-01622-s001.zip › Document S7.pdf]

**Por favor, lea con atención las siguientes preguntas y luego contéstelas:**

1. ¿Le dio usted Lactancia Materna a su hijo/a? (Si responde No, pase a la pregunta 4).
   - ☐ Si
   - ☐ No
2. ¿Cuánto tiempo le dio usted Lactancia materna? Ya sea sola o combinada.
   - ☐ Menos de un mes. Especifique por favor en días o semanas

     Días: _____________

     Semanas: _________________

   - ☐ Un mes
   - ☐ Dos meses
   - ☐ Tres meses
   - ☐ Cuatro meses
   - ☐ Cinco meses
   - ☐ Seis meses
   - ☐ Siete meses
   - ☐ Ocho meses
   - ☐ Nueve meses
   - ☐ Diez meses
   - ☐ Once meses
   - ☐ Un año
   - ☐ Más de un año: Para este caso especifique por favor: 1 año y _____ meses
   - ☐ Dos años
   - ☐ Más de dos años: Para este caso especifique por favor: 2 años y _____ meses
   - ☐ Tres años
   - ☐ Más de tres años: Para este caso especifique por favor: 3 años y _____meses
   - ☐ Cuatro años
   - ☐ Más de cuatro años: Para este caso especifique por favor: 4 años y _____ meses

3. ¿Cuánto tiempo le dio usted SOLO lactancia materna? Es decir, indíquenos por favor cuanto tiempo (días, semanas o meses) que su hijo/a se alimentó solamente con leche materna.

4. ¿Cuándo introdujo usted por primera vez un alimento diferente a la leche (zumo, galletas, cereales…)?
   - ☐ Antes del primer mes
   - ☐ A partir del primer mes
   - ☐ A partir del segundo mes
   - ☐ A partir del tercer me
   - ☐ A partir del cuarto mes
   - ☐ A partir del quinto mes
   - ☐ A partir del sexto mes
   - ☐ No lo recuerdo

5. Su hijo/a nació
   - ☐ Por parto natural
   - ☐ Por cesárea
6. Indique por favor si lo recuerda, el peso de su hijo/a al nacer:

7. Su hijo/a nació:
   - ☐ A término (es decir, entre la semana 37 y 42)
   - ☐ Prematuramente (antes de la semana 37)

**¡¡MUCHAS GRACIAS POR SU PARTICIPACIÓN!!**
