# Supplementary material for: A Multimodal Intervention for Prevention of Overweight and Obesity in Schoolchildren. A Protocol Study “PREVIENE-CÁDIZ”
Source: Int J Environ Res Public Health. 2021 Feb 8;18(4):1622. doi: 10.3390/ijerph18041622 (PMC7914756; doi:10.3390/ijerph18041622)
Supplement: Supplementary file 1 [file ijerph-18-01622-s001.zip › Document S8.pdf]

Universidad de Cádiz · Junta de Andalucía · Escuela Andaluza de Salud Pública · CONSEJERÍA DE SALUD · Junta de Andalucía Consejería de Educación Delegación Provincial de Cádiz · Junta de Andalucía · Unión Europea Fondo Europeo de Desarrollo Regional · Andalucía se mueve con Europa

Cuestionario de Evaluación del Programa *PREVIENE CÁDIZ*.

Directores/as – Equipos directivos

Previene Cádiz

Gracias por participar en este proyecto. Su opinión es muy importante.

Por favor cumplimente este formulario que nos permitirá evaluar el funcionamiento del Programa y mejorarlo con sus propuestas.

La duración prevista es de 5 minutos.

Hay 14 preguntas en la encuesta.

Nombre del centro ______

Localidad ______

Persona que responde la encuesta:

**Director/a del centro**		
**Otra persona**		**Indicar quien:**

1. ¿Considera interesante incluir en el Programa *PREVIENE CÁDIZ* alguna otra actividad relacionada con los contenidos abordados?

Por favor seleccione **sólo una** de las siguientes opciones:

- ○ Sí
- ○ No

Descríbala brevemente. (Sólo conteste esta pregunta si la respuesta fue "Sí" en la pregunta anterior).

Por favor, escriba su respuesta aquí:

______

2. Valore la implicación de las familias con el desarrollo del programa.

Valore con una escala del 1 al 5 la implicación de las familias en el programa, donde 1 significa que *no se han implicado nada* y 5 que *se han implicado mucho*.

Por favor seleccione **sólo una** de las siguientes opciones:

No se han implicado nada [1] [2] [3] [4] [5] Se han implicado mucho

3. ¿Cree que el profesorado necesita más recursos para llevar a cabo el programa PREVIENE-Cádiz en las áreas que se especifican a continuación?

Por favor, marque las opciones que correspondan:

- ☐ Formación
- ☐ Materiales
- ☐ Apoyo de profesionales externos/as
- ☐ Tiempo para desarrollar las sesiones
- ☐ Otro: ____________________

4. ¿Volvería a participar en este programa el próximo curso escolar?

Por favor, seleccione **sólo una** de las siguientes opciones:

- ○ Sí
- ○ No

5. ¿Recomendaría este programa a otros centros?

Por favor, seleccione **sólo una** de las siguientes opciones:

- ○ Sí
- ○ No

6. ¿Se han aplicado los recreos activos en su centro?

Por favor, seleccione **sólo una** de las siguientes opciones:

- ○ Sí
- ○ No

7. Señale el motivo principal por el que no se llevaron a cabo los recreos activos.

Sólo conteste esta pregunta si la respuesta fue 'No' en la pregunta 25. Por favor, seleccione **sólo una** de las siguientes opciones:

- ○ No sabía cómo aplicar la propuesta de recreos activos.
- ○ Creo que la propuesta de recreos activos no es eficaz.
- ○ Considero que el alumnado ya es lo suficientemente activo durante los recreos.
- ○ No estaba dispuesto/a a dedicar más tiempo al programa.
- ○ Otro. Indíquelo: ____________________

8. ¿Podría aportar alguna sugerencia que, desde su punto de vista, contribuyera a mejorar el programa?

Por favor, escriba su respuesta aquí:

9. ¿Se ha desarrollado en su centro este año el programa "Creciendo en salud"? Sí ☐ No ☐ No lo sé ☐

10. En caso negativo ¿nos podría indicar cuales son las causas de no haberlo desarrollado?

11. En caso afirmativo, indicar qué líneas del mismo se han desarrollado: (en 3er curso)

Educación emocional.	
Estilos de vida Saludable.	
Autocuidados y Accidentalidad.	
Uso Positivo de las Tecnologías de la Información y la Comunicación	
Prevención del Consumo de Sustancias Adictivas.	

12. En el caso de que se haya desarrollado alguna/s de las líneas anteriores, indicar qué bloques temáticos se han trabajado: (en 3er curso)

Educación emocional.	
- Conciencia Emocional.	
- Regulación Emocional.	
- Autonomía Emocional.	
- Competencia Social.	
- Competencias para la Vida y el Bienestar.	
Estilos de vida Saludable.	
- Actividad Física.	
- Alimentación Equilibrada.	
Autocuidados y Accidentalidad.	
- Autocuidados	
- Accidentalidad:	
o Educación Vial	
o Seguridad en el Hogar	
Uso Positivo de las Tecnologías de la Información y la Comunicación	
- Estilos de Vida Saludable en una Sociedad digital.	
- Adicciones a las TIC.	
- Buenas Prácticas y Recomendaciones.	
Prevención del Consumo de Sustancias Adictivas.	
- Hábitos que contribuyen a una vida sana.	
- Consumo de Alcohol y Tabaco.	
- Publicidad y Drogas: mitos y creencias.	

13. ¿Disponéis en el Centro de un equipo de Promoción y Educación para la Salud? (Consejo de Salud Escolar o similar, grupo de personas encargados de estas actividades) Si ☐ No ☐

En caso afirmativo,

¿Quién lo ha coordinado?____

¿Quién lo compone? (indicar las personas que lo componen, sus cargos y/o colectivos a los que representan, no es necesario indicar nombres y apellidos)

14. Actividades realizadas este curso académico en el centro educativo con el alumnado de 3º curso, relacionadas con la alimentación y/o el ejercicio físico saludables, <u>además de las incluidas en el programa PREVIENE</u>.

Plan Escolar de Consumo de Frutas y Hortalizas (reparto de frutas / hortalizas)	
Desayuno saludable.	
Concurso de recetario infantil.	
Huertos escolares.	
Frutibus	
Otras:	

Indicar qué otras actividades se han realizado:

Muchas gracias por completar el cuestionario.
