# Supplementary material for: A Multimodal Intervention for Prevention of Overweight and Obesity in Schoolchildren. A Protocol Study “PREVIENE-CÁDIZ”
Source: Int J Environ Res Public Health. 2021 Feb 8;18(4):1622. doi: 10.3390/ijerph18041622 (PMC7914756; doi:10.3390/ijerph18041622)
Supplement: Supplementary file 1 [file ijerph-18-01622-s001.zip › Document S9.pdf]

Cuestionario de Evaluación del Programa *PREVIENE CÁDIZ*.

Docentes

Gracias por participar en este proyecto. Su opinión es muy importante.

Por favor cumplimente este formulario que nos permitirá evaluar el funcionamiento del Programa y mejorarlo con sus propuestas.

La duración prevista es de 15 minutos.

Hay 35 preguntas en la encuesta.

Nombre del centro [ ]

Localidad [ ]

Señale con una X la que corresponda.

Línea | 1 | 2 | 3 | 4 |

Persona que responde la encuesta:

**Profesor/a tutor/a de la línea**			
**Profesor/a de Educación Física**			
**Otro/a:**		**Indicar quien:**	

1. Indique la fecha aproximada en la que comenzó la primera actividad o primera sesión del programa PREVIENE (fecha en que comenzó el programa) ___ /___ /___

2. Indique la fecha aproximada en la que terminó la última actividad o última sesión del programa PREVIENE (fecha en que terminó el programa) ___ /___ /___

3. ¿Considera interesante incluir en el Programa *PREVIENE CÁDIZ* alguna otra actividad relacionada con los contenidos abordados?

Por favor seleccione **sólo una** de las siguientes opciones:

○ Sí

○ No

Descríbala brevemente.

(Sólo conteste esta pregunta si la respuesta fue "Sí" en la pregunta anterior).

Por favor, escriba su respuesta aquí:

[ ]

4. Señale si ha desarrollado el programa según la Guía Didáctica.

Por favor, seleccione con una X la respuesta apropiada para cada concepto:

	Realizada según programa	No realizada	Realizada de forma alternativa
Sesión 1	○	○	○
Sesión 2	○	○	○
Sesión 3	○	○	○
Sesión 4	○	○	○
Sesión 5	○	○	○
Sesión 6	○	○	○
Sesión 7	○	○	○
Sesión 8	○	○	○
Sesión 9	○	○	○
Foto y Voz	○	○	○

5. En el caso de que en la pregunta 4 haya indicado que la sesión 1 la ha desarrollado de forma alternativa, indique las modificaciones realizadas (material de apoyo, técnicas empleadas, tiempo dedicado, etc.).

Sesión 1. Por favor, escriba su respuesta aquí:

6. En el caso de que en la pregunta 4 haya indicado que la sesión 2 la ha desarrollado de forma alternativa, indique las modificaciones realizadas (material de apoyo, técnicas empleadas, tiempo dedicado, etc.).

Sesión 2. Por favor, escriba su respuesta aquí:

7. En el caso de que en la pregunta 4 haya indicado que la sesión 3 la ha desarrollado de forma alternativa, indique las modificaciones realizadas (material de apoyo, técnicas empleadas, tiempo dedicado, etc.).

Sesión 3. Por favor, escriba su respuesta aquí:

8. En el caso de que en la pregunta 4 haya indicado que la sesión 4 la ha desarrollado de forma alternativa, indique las modificaciones realizadas (material de apoyo, técnicas empleadas, tiempo dedicado, etc.).

Sesión 4. Por favor, escriba su respuesta aquí:

9. En el caso de que en la pregunta 4 haya indicado que la sesión 5 la ha desarrollado de forma alternativa, indique las modificaciones realizadas (material de apoyo, técnicas empleadas, tiempo dedicado, etc.).

Sesión 5. Por favor, escriba su respuesta aquí:

10. En el caso de que en la pregunta 4 haya indicado que la sesión 6 la ha desarrollado de forma alternativa, indique las modificaciones realizadas (material de apoyo, técnicas empleadas, tiempo dedicado, etc.).

Sesión 6. Por favor, escriba su respuesta aquí:

11. En el caso de que en la pregunta 4 haya indicado que la sesión 7 la ha desarrollado de forma alternativa, indique las modificaciones realizadas (material de apoyo, técnicas empleadas, tiempo dedicado, etc.).

Sesión 7. Por favor, escriba su respuesta aquí:

12. En el caso de que en la pregunta 4 haya indicado que la sesión 8 la ha desarrollado de forma alternativa, indique las modificaciones realizadas (material de apoyo, técnicas empleadas, tiempo dedicado, etc.).

Sesión 8. Por favor, escriba su respuesta aquí:

13. En el caso de que en la pregunta 4 haya indicado que la sesión 9 la ha desarrollado de forma alternativa, indique las modificaciones realizadas (material de apoyo, técnicas empleadas, tiempo dedicado, etc.).

Sesión 9. Por favor, escriba su respuesta aquí:

14. En el caso de que en la pregunta 4 haya indicado que el Foto y Voz lo ha desarrollado de forma alternativa, indique las modificaciones realizadas (material de apoyo, técnicas empleadas, tiempo dedicado, etc.).

Foto y Voz. Por favor, escriba su respuesta aquí:

15. Valoración de las sesiones:

Señale con una X aquellas contestaciones afirmativas (SÍ).

	Sesión 1	Sesión 2	Sesión 3	Sesión 4	Sesión 5	Sesión 6	Sesión 7	Sesión 8	Sesión 9	FotoVoz
La participación del alumnado fue buena	☐	☐	☐	☐	☐	☐	☐	☐	☐	☐
Los contenidos fueron comprendidos	☐	☐	☐	☐	☐	☐	☐	☐	☐	☐
Los materiales fueron usados satisfactoriamente	☐	☐	☐	☐	☐	☐	☐	☐	☐	☐

16. Valore la implicación de las familias con el desarrollo del programa.

Valore con una escala del 1 al 5 la implicación de las familias en el programa, donde 1 significa que *no se han implicado nada* y 5 que *se han implicado mucho*.

Por favor seleccione **sólo una** de las siguientes opciones:

No se han implicado nada ☐ 1 ☐ 2 ☐ 3 ☐ 4 ☐ 5 Se han implicado mucho

17. De los materiales del programa, indique cuáles ha usado.

Por favor, seleccione con una X la respuesta apropiada para cada concepto:

	Sí	No
Guía didáctica del profesorado	○	○
Material alumnado	○	○
Material familias	○	○

18. Valore los siguientes aspectos de la Guía didáctica destinada al profesorado:

Valore dichos aspectos con una escala del 1 al 5, donde 1 se corresponde con una puntuación muy baja y 5 con una puntuación muy alta.

	Sesión 1	Sesión 2	Sesión 3	Sesión 4	Sesión 5	Sesión 6	Sesión 7	Sesión 8	Sesión 9	Foto Voz*
Claridad, comprensión y legibilidad del contenido por parte del docente	☐	☐	☐	☐	☐	☐	☐	☐	☐	☐
Idoneidad de la metodología propuesta	☐	☐	☐	☐	☐	☐	☐	☐	☐	☐
Coherencia entre los objetivos, contenidos y actividades	☐	☐	☐	☐	☐	☐	☐	☐	☐	☐

	Sesión 1	Sesión 2	Sesión 3	Sesión 4	Sesión 5	Sesión 6	Sesión 7	Sesión 8	Sesión 9	Foto Voz*
Calidad del diseño y las imágenes	☐	☐	☐	☐	☐	☐	☐	☐	☐	☐
Ajuste de la sesión al tiempo programado	☐	☐	☐	☐	☐	☐	☐	☐	☐	☐

*Información aportada de manera complementaria a la Guía Didáctica

19. Valore los siguientes criterios del material para el alumnado.

Por favor, seleccione con una X la respuesta apropiada para cada concepto, donde 1 se corresponde con una puntuación muy baja y 5 con una puntuación muy alta.

	1	2	3	4	5
Claridad, comprensión y legibilidad por parte del alumnado	○	○	○	○	○
Calidad del diseño y las imágenes	○	○	○	○	○

20. Valore los siguientes criterios del material para las familias.

Por favor, seleccione con una X la respuesta apropiada para cada concepto, donde 1 se corresponde con una puntuación muy baja y 5 con una puntuación muy alta.

	1	2	3	4	5
Claridad, comprensión y legibilidad por parte de las familias	○	○	○	○	○
Calidad del diseño y las imágenes	○	○	○	○	○

21. ¿Cree que el profesorado necesita más recursos para llevar a cabo el programa en las áreas que se especifican a continuación?

Por favor, marque las opciones que correspondan:

☐ Formación

☐ Materiales

☐ Apoyo de profesionales externos/as

☐ Tiempo para desarrollar las sesiones

☐ Otro: ______________________

22. Identifique las principales dificultades que ha encontrado para desarrollar las sesiones.

Por favor, marque las opciones que correspondan:

☐ Falta de tiempo para desarrollar la intervención completa (9 sesiones)

☐ El tiempo previsto para cada sesión no se ajusta a la duración de una clase

☐ Realización paralela de otros programas

☐ Algunos contenidos ya se han trabajado en otras materias

☐ Otro: ______________________

23. ¿Volvería a participar en este programa el próximo curso escolar?

Por favor, seleccione **sólo una** de las siguientes opciones:

◯ Sí

◯ No

24. ¿Recomendaría este programa a otros/as docentes?

Por favor, seleccione **sólo una** de las siguientes opciones:

◯ Sí

◯ No

25. ¿Se han aplicado los recreos activos en su centro?

Por favor, seleccione **sólo una** de las siguientes opciones:

◯ Sí

◯ No

26. ¿En qué nivel se llegó a aplicar?

Sólo conteste esta pregunta si la respuesta fue 'Sí' en la pregunta 25. Por favor, seleccione **sólo una** de las siguientes opciones:

◯ 1. El/la docente imprimió y colocó la hoja de control en un lugar visible y accesible al alumnado.

◯ 2. El maestro o maestra, además de lo anterior, controló que el alumnado anotase en la hoja de control cada día que realizaban actividad física durante el recreo.

◯ 3. El/la docente, además de lo anterior (1+2) informó al alumnado sobre lo que significa ser activo/a durante el recreo y les animó a participar.

◯ 4. El/la docente, además de todo lo anterior (1+2+3) hizo que el alumnado propusiese al menos tres actividades o juegos activos para realizar en los recreos.

◯ 5. El maestro o maestra, además de todo lo anterior (1+2+3+4), colocó en la pared de la clase un póster o cartel modelo con una frase y dibujo saludable.

27. En caso de que el alumnado elaborase póster o carteles o alguna actividad, indíquela por favor.

Sólo conteste esta pregunta si la respuesta fue 'Sí' en la pregunta 25. Por favor, marque las opciones que correspondan:

☐ Cada alumno/a realizó un póster o cartel en el aula o en casa con la familia, y lo mostró en clase.

☐ Se realizó un concurso con los posters o carteles realizados por el alumnado, eligiendo entre ellos/as los mejores.

☐ Se realizó un Collage saludable entre el alumnado, aportando cada uno/a una frase o un dibujo de contenido de salud.

☐ Se realizó algún tipo de representación (rap, teatro,...) de contenido saludable en grupos reducidos.

☐ Se realizó alguna actividad que logró implicar a todo el centro (profesorado y alumnado) (p.e. un Lipdub, Flashmob).

☐ Otra propuesta. Indíquela: ______________________

28. Señale el motivo principal por el que no se llevaron a cabo los recreos activos.

Sólo conteste esta pregunta si la respuesta fue 'No' en la pregunta 25. Por favor, seleccione **sólo una** de las siguientes opciones:

- ◯ No sabía cómo aplicar la propuesta de recreos activos.
- ◯ Creo que la propuesta de recreos activos no es eficaz.
- ◯ Considero que el alumnado ya es lo suficientemente activo durante los recreos.
- ◯ No estaba dispuesto/a a dedicar más tiempo al programa.
- ◯ Otro. Indíquelo:

29 ¿Podría aportar alguna sugerencia que, desde su punto de vista, contribuyera a mejorar el programa?

Por favor, escriba su respuesta aquí:

30. ¿Se ha desarrollado en tu centro este año el programa "Creciendo en salud"? Sí ☐ No ☐ No lo sé ☐

31. En caso negativo ¿nos podrías indicar cuales son las causas de no haberlo desarrollado?

32. En caso afirmativo, indicar qué líneas del mismo se han desarrollado: (en 3er curso)

Educación emocional.	
Estilos de vida Saludable.	
Autocuidados y Accidentalidad.	
Uso Positivo de las Tecnologías de la Información y la Comunicación	
Prevención del Consumo de Sustancias Adictivas.	

33. En el caso de que se haya desarrollado alguna/s de las líneas anteriores, indicar qué bloques temáticos se han trabajado: (en 3er curso)

Educación emocional.	
- Conciencia Emocional.	
- Regulación Emocional.	
- Autonomía Emocional.	
- Competencia Social.	
- Competencias para la Vida y el Bienestar.	
Estilos de vida Saludable.	
- Actividad Física.	
- Alimentación Equilibrada.	
Autocuidados y Accidentalidad.	
- Autocuidados	
- Accidentalidad:	
o Educación Vial	
o Seguridad en el Hogar	
Uso Positivo de las Tecnologías de la Información y la Comunicación	
- Estilos de Vida Saludable en una Sociedad digital.	
- Adicciones a las TIC.	
- Buenas Prácticas y Recomendaciones.	
Prevención del Consumo de Sustancias Adictivas.	
- Hábitos que contribuyen a una vida sana.	
- Consumo de Alcohol y Tabaco.	
- Publicidad y Drogas: mitos y creencias.	

34. ¿Disponéis en el Centro de un equipo de Promoción y Educación para la Salud? (Consejo de Salud Escolar o similar, grupo de personas encargados de estas actividades) Si ☐ No ☐

En caso afirmativo,

¿Quién lo ha coordinado?____

¿Quién lo compone? (indicar las personas que lo componen, sus cargos y/o colectivos a los que representan, no es necesario indicar nombres y apellidos)

35. Actividades realizadas este curso académico en el centro educativo con el alumnado de 3º curso, relacionadas con la alimentación y/o el ejercicio físico saludables, <u>además de las incluidas en el programa PREVIENE</u>.

Plan Escolar de Consumo de Frutas y Hortalizas (reparto de frutas / hortalizas)	
Desayuno saludable.	
Concurso de recetario infantil.	
Huertos escolares.	
Frutibus	
Otras:	

Indicar qué otras actividades se han realizado:

Muchas gracias por completar el cuestionario.
